# Burden of varicella in Latin America and the Caribbean: findings from a systematic literature review

**DOI:** 10.1186/s12889-019-6795-0

**Published:** 2019-05-08

**Authors:** Luiza Helena Falleiros Arlant, Maria Catalina Pirez Garcia, Maria L. Avila Aguero, Miguel Cashat, Cintia Irene Parellada, Lara J. Wolfson

**Affiliations:** 1 Universidade Metropolitana de Santos – UNIMES – Santos, São Paulo, Brazil; 2 Universidad De La Republica, Montevideo, Uruguay; 3 National Children’s Hospital Costa Rica, San Jose, Costa Rica; 4 Affiliated Researcher Center for Infectious Disease Modeling and Analysis (CIDMA) at Yale University, New Haven, CT USA; 5 Global Medical Affairs, MSD México, México City, México; 6 Center for Observational and Real-World Evidence, MSD Brazil, São Paulo, Brazil; 7 Center for Observational and Real-World Evidence (CORE), Merck & Co., Inc., Kenilworth, NJ 07033 USA

**Keywords:** Caribbean, Latin America, Systematic literature review, Vaccination, Varicella

## Abstract

**Background:**

Varicella is typically mild and self-limiting, but can be associated with complications and even death. The limited data available on varicella in Latin America and the Caribbean (LAC) indicate substantial burden in countries where varicella vaccine is not part of publicly funded childhood national immunization programs.

**Methods:**

A systematic literature review of published studies was complemented by “gray” literature on varicella incidence, complications, mortality, and economic consequences, in the absence and presence of universal varicella vaccination (UVV) in LAC.

**Results:**

Seroprevalence data indicate that varicella is usually a disease of childhood in LAC. Varicella incidence rates, while unreliable in the absence of mandatory reporting, show a trend to increased incidence due to greater urbanization and population density. The introduction of UVV in national immunization programs has led to significant reductions in varicella incidence in these areas.

**Conclusions:**

Varicella continues to pose a substantial healthcare burden in LAC. The future introduction of UVV in additional countries is predicted to provide substantial reductions in cases, with important economic benefits. For countries that have already implemented UVV, the challenge is to maintain high rates of coverage and, where relevant, consider inclusion of a second dose to reduce breakthrough cases. Given the significant proportion of the region now implementing UVV, a regional recommendation in order to prevent any potential for age-shifts in varicella infection might be considered.

**Electronic supplementary material:**

The online version of this article (10.1186/s12889-019-6795-0) contains supplementary material, which is available to authorized users.

## Background

Varicella (chickenpox) is an acute, common, and highly contagious infection caused by the varicella-zoster virus (VZV), a human herpesvirus [1]. VZV is transmitted by direct contact and by inhalation of aerosols from vesicular fluid of skin lesions of varicella or herpes zoster. Typically, varicella is a mild and self-limiting disease, characterized by a generalized itchy rash, headache, fever, and malaise [2, 3]. However, serious complications can occur, involving the central nervous system, respiratory system, or skin, and occasionally leading to death; adults, young infants, and immunocompromised individuals are at higher risk for severe complications [1]. Based on conservative estimates, varicella is responsible for 4.2 million severe complications leading to hospitalization and for 4200 deaths worldwide each year [4].

The epidemiology of varicella varies globally. In high-income countries with a temperate climate, varicella is usually a childhood disease, showing seasonal variation; in the pre-vaccination era, more than 90% of infections occurred before adolescence, with fewer than one in 20 adults remaining susceptible [4]. In some tropical countries, by contrast, for reasons still unclear, the average age of infection is higher, with adolescents and adults showing increased susceptibility compared with temperate regions [5–7]. Higher population density, which can be associated with increased urbanization, and early-age schooling are also related to elevated varicella incidence [8, 9]. Latin America and the Caribbean (LAC) is a diverse region of mainly middle-income countries, mostly located within the tropics. Compared with the United States and other high-income, temperate countries, the data on varicella epidemiology in LAC are scarce [10, 11].

Varicella is a vaccine-preventable disease and immunization using live attenuated varicella vaccine provides effective and long-lasting protection [1, 12]. There are two forms of the varicella vaccine: a single-component vaccine and a multicomponent vaccine, i.e. the combination quadrivalent measles, mumps, rubella, varicella (MMRV) vaccine. The major global experience of vaccine effectiveness is based on OKA strain varicella vaccines. Based on a systematic review of all publications on varicella vaccine effectiveness from 1995 to 2014, the effectiveness for one dose of OKA-strain varicella vaccines was estimated to be 81% (95% confidence interval [CI]: 78, 84%) against all varicella, and 98% (95% CI: 97, 99%) against moderate/severe varicella [13]. The pooled two-dose estimate of vaccine effectiveness of OKA-strain varicella vaccines against all varicella was 92% (95% CI: 88, 95%) [13]. Introduction of OKA-strain varicella vaccines has led to a dramatic decline in the morbidity and mortality associated with varicella – for example, an 81–88% decline in varicella-related hospitalizations in Uruguay, Canada and the United States [14–17] and an 88% decline in varicella-associated deaths in the United States [14].

**Fig. 1:**
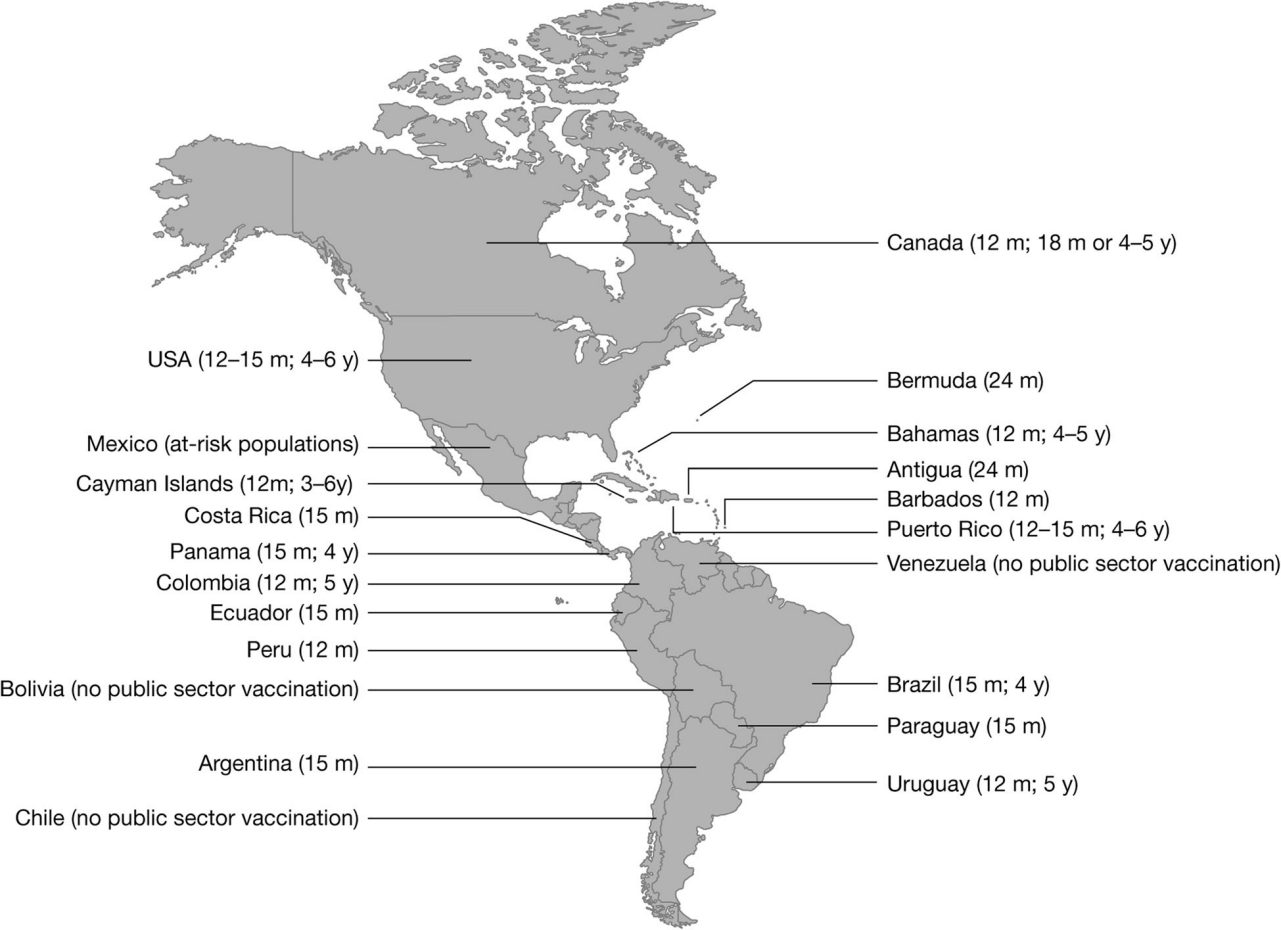
Countries in Latin and North America that introduced varicella vaccination, with ages of administration

The World Health Organization recommends that countries with a substantial public health burden of varicella should consider incorporating vaccination within the routine childhood immunization program, provided resources can support ≥ 80% coverage [4]. In 2016, the Latin American Society of Pediatric Infectious Diseases (Sociedad Latinoamericana de Infectologia Pediátrica [SLIPE]) recommended the inclusion of two-dose varicella vaccination within the national immunization schedules of all LAC countries [10, 18]. As of January 2018, 14 (out of 35) countries had implemented universal varicella vaccination (UVV), including six with a two-dose schedule [19] (Table 1 and Fig. 1). In February 2018, Peru initiated UVV, and Brazil is in the process of transitioning from a one- to a two-dose schedule [20, 21]. Currently, monovalent varicella vaccines (both OKA-strain and MAV-strain) are used throughout LAC where publicly-funded varicella immunization programs are in place. In Brazil, both monovalent (MAV and/or OKA) and MMRV (OKA only) vaccines are administered as the first dose at 15 months old. Currently, 80% of each birth cohort in South America, but only 20% of each birth cohort in Central America and the Caribbean, have access to UVV.

**Table 1:** Universal varicella vaccine in national immunization programs in LAC

Country	First Dose		Second Dose		Type of Varicella Vaccine^a^
	Year Introduction	Age, mo	Year Introduction	Age, y	
Antigua	2014	24	–	–	Monovalent
Argentina	2015	15	–	–	Monovalent
Bahamas	2012	12	2012	4–5	Monovalent
Barbados	2012	12	–	–	Monovalent
Bermuda	2012	24	–	–	Monovalent
Brazil	2013	15	2018	4	MMRV/Monovalent
Cayman Islands	2000	12	2009	3–6	Monovalent
Colombia	2015	12	2019^b^	5	Monovalent
Costa Rica	2007	15	–	–	Monovalent
Ecuador	2010	15	–	–	Monovalent
Panama	2014	15	2018	4	Monovalent
Paraguay	2013	15	–	–	Monovalent
Peru	2018	12	–	–	Monovalent
Puerto Rico	1996	12–15	2007	4–6	Monovalent
Uruguay	1999	12	2014	5	Monovalent

*LAC* Latin America and the Caribbean; *MMRV* measles, mumps, rubella, and varicella vaccine

^a^Monovalent: varicella-only vaccine

^b^Colombia plans to start the second dose in 2019, when the cohort vaccinated at 12 months reaches 5 years old

Coverage rates among the target population in LAC that introduced UVV have tended to be high soon after implementation. In Costa Rica, where UVV was introduced in September 2007, coverage increased from 76% in 2008 to 95% in 2015 [22]. In Uruguay, where UVV was introduced in 1999, 90% coverage with the one-dose schedule was achieved rapidly, and was maintained up to the point of data publication (2008, 2013) [15, 23]. Other countries have targeted publicly funded vaccination to at-risk groups instead of adopting universal vaccination. For example, in Mexico, publicly funded vaccination is provided to children attending child development centers, people with immunodeficiency, children with cancer, and certain susceptible healthcare workers (HCWs) [10].

Local data from LAC on varicella incidence, complications, mortality, and economic consequences are needed to better understand the disease burden and the actual or potential effects of vaccination in the region. This review aims to describe trends relevant to improving the understanding, prevention, and management of varicella across LAC and is part of a broader systematic literature review (SLR), undertaken to assess the epidemiology and burden of varicella infection and the status of varicella vaccination programs in global regions.

## Methods

The methodology adopted in this SLR follows the Preferred Reporting Items for Systematic Reviews and Meta-Analysis (PRISMA) guidelines [24] and includes a protocol with predefined objectives (described above) as well as eligibility criteria for the inclusion of studies (see Additional file 1).

### Eligibility criteria for studies

Studies were eligible for inclusion in the SLR if they: (1) included males or females of any age and race who had primary and/or breakthrough varicella infection or were undergoing serological testing for antibodies to varicella; (2) assessed the epidemiological and/or economic burden of varicella infection; and (3) were of the following study design or study type: epidemiological, cohort, case-control, cross-sectional, or registry/database. Cost studies/surveys/analyses, budget impact models, database cost studies, resource-use studies, or cost of illness studies; cost-effectiveness, cost-utility, cost-benefit, cost-minimization, and cost-consequence analyses; and routine surveillance reports were also included.

### Information sources

Embase® and MEDLINE® biomedical databases were searched (using Embase.com platform) for publications from inception up to 1 February 2016. Regional databases, including the Latin American and Caribbean Health Sciences Information database (LILACS; produced by BIREME, Latin American and Caribbean Center on Health Sciences Information; http://lilacs.bvsalud.org/en/), were also searched. In addition to English-language publications, scientific leaders in LAC (coauthors in this publication) also identified important local studies published in Spanish or Portuguese for inclusion, up to the present (2018). Bibliographies of systematic reviews and meta-analyses identified through database searches were also examined for additional eligible studies.

Gray literature (i.e. not formally published) and additional sources were searched to address data gaps, including Ministry of Health websites for LAC countries, World Health Organization and Pan American Health Organization (PAHO) websites, SLIPE data review/position papers, and congress presentations (as cited below).

### Database search strategy

Separate searches were conducted on Embase® and MEDLINE® for eligible studies that assessed the epidemiology, economic burden, and vaccination program status (see Additional file 2). The LILACS database was searched using the following terms: chickenpox OR varicella OR chicken pox [title words] or chickenpox OR varicella OR chicken pox [abstract words] and not herpes [title words].

### Study selection

Publications identified through electronic database searches were initially screened for inclusion based on title and abstract. Full-text copies of studies that were potentially eligible were then screened. Screening was undertaken by a single reviewer. A second independent reviewer validated a random sample of 20% of studies from both first (title/abstract) and second (full-text) screening.

### Data collection

Data from the studies identified through database searches were extracted into a predefined extraction grid by a single reviewer, and subsequently validated by a second independent reviewer. Studies with multiple publications were extracted into a single entry. Data for parameters including the following were extracted: publication type and citation information; study information (region, country, objective, setting, design, data collection period, patient population, age and sex, and sample size); study conclusions; reported outcomes (epidemiology, vaccination program, resource use, economic burden, or economic evaluation); and main methodology and outcomes data.

### Currency conversion for economic measures

Cost data were adjusted to 2017 US dollars by initially using country-specific annual inflation rates to obtain 2017 costs in country-specific currencies and, subsequently, converting all costs to US dollars based on exchange rates using country-specific websites (CCEMG - EPPI-Centre Cost Converter at https://eppi.ioe.ac.uk/costconversion/default.aspx and the CPI Inflation Calculator at https://www.bls.gov/data/inflation_calculator.htm). For studies where cost-year was not mentioned, the publication year was considered as the cost-year for all calculations.

## Results

### Study selection and characteristics

**Fig. 2:**
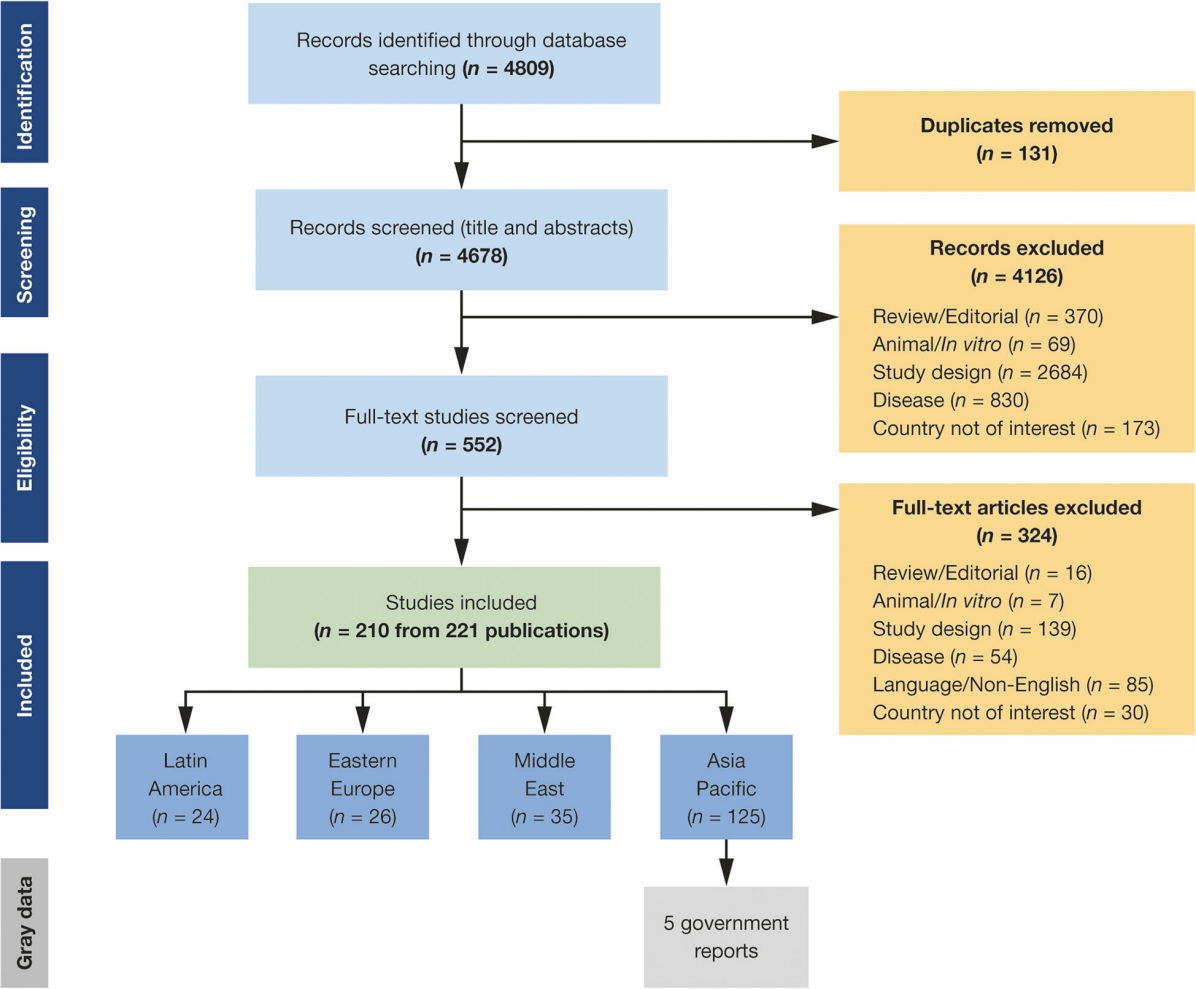
PRISMA flow chart showing study selection for the LAC and other regions studied

A total of 4678 global records were identified from literature database searches and screened. Of these, the full texts of 552 studies were assessed for eligibility, resulting in 210 studies (221 publications) for inclusion in the overarching SLR across the four global regions, including LAC (Fig. 2); 24 of the identified studies were relevant to LAC.

The studies from the LAC region included data from the following countries, categorized by income strata (World Bank 2017–2018): Bolivia (lower-middle-income economy); Argentina, Brazil, Colombia, Costa Rica, Ecuador, Mexico, Panama, Paraguay, Peru, St Lucia, and Venezuela (upper-middle-income); and Bahamas, Barbados, Chile, Puerto Rico, and Uruguay (high-income) (Table 2).

**Table 2:** Studies identified from the SLR in the LAC region

Study type	Countries with data available
Epidemiology burden	Argentina [25, 26]
	Bolivia [6, 7]
	Brazil [27–36]
	Colombia [37, 38]
	Costa Rica [22]
	Mexico [39–42]
	Puerto Rico [43]
	Uruguay [15]
Varicella vaccination program	Argentina [44]
	Brazil, [33, 45]
	Costa Rica [22]
	Mexico [41, 42]
	Uruguay [15]
Economic burden	Argentina [26]
	Brazil [28, 31–33]
	Colombia [37]
	Costa Rica [22]
	Uruguay [15]

*SLR* systematic literature review; *LAC* Latin America and the Caribbean

Scientific leaders in LAC identified a further six published studies from LAC for inclusion [23, 46–50]. Additional relevant SLIPE position papers, gray literature sources, and congress abstracts/posters are also discussed in the sections that follow.

### Epidemiological burden of varicella infection

In total, 23 studies from the SLR provided evidence on the epidemiology of varicella in LAC. The sample size of these observational studies ranged from 75 to 294,831 patients. The most commonly reported outcome was seroprevalence, followed by incidence, mortality, and complications. Study data from these papers are summarized in Additional file 3.

### Seroprevalence

A total of 12 published studies provided evidence on the seroprevalence of VZV in LAC (see Additional file 3).

Among population-based studies of children and adults, in the absence of or before the introduction of routine varicella vaccination, the overall seroprevalence rate ranged between 58 and 99% in LAC: 58% in Puerto Rico [43], 78–80% in Bolivia [6, 7], 81% among Xingu indigenous ethnic groups in Brazil [27], 82% among Montevideo inhabitants in Uruguay [50], 86–99% in nationally representative samples in Mexico [39, 40], and 97.2–99.3% in different parts of Argentina [25].

The only factors consistently associated with seroprevalence rate were age and reported history of varicella infection [25, 27–29].

**Fig. 3:**
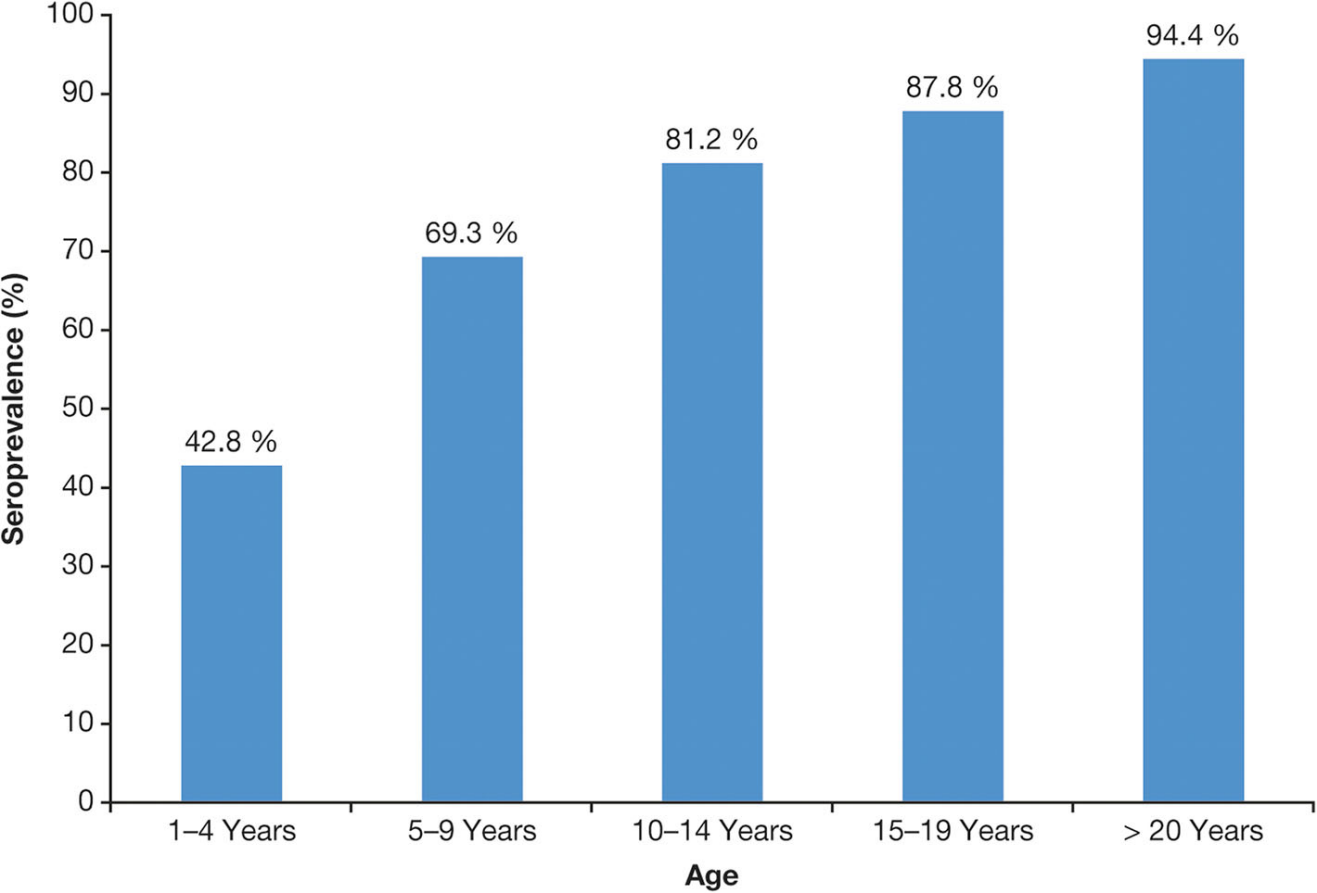
Seroprevalence of VZV in different age groups in Mexico, 2005 and 2006 [39]

**Fig. 4:**
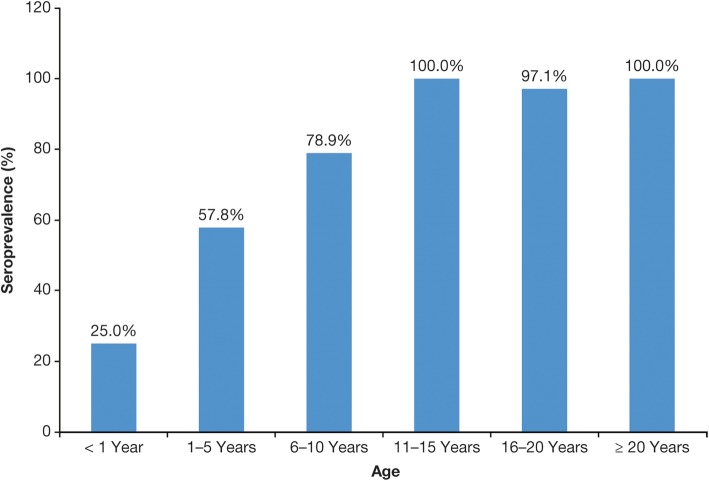
Seroprevalence of VZV in different age groups in Brazil in 2001 [27]

The seroprevalence data show that varicella in most of LAC is typically a disease of childhood rather than adolescence or adulthood and, in this regard, is generally closer to temperate countries before UVV programs were introduced. Seroprevalence rates in LAC consistently increase with age [6, 7, 25, 27, 29, 39, 40]. In a study in Mexico, for example, seroprevalence rates were 69% in children aged 5–9 years, rising to 94% in adults ≥ 20 years [39] (Fig. 3). In a Brazilian study, seroprevalence increased with age up to the 11- to 15-year age group, when it attained 100% [27] (Fig. 4).

Additional factors identified to influence seroprevalence rate in some studies included the level of crowded living conditions [25], extent of school age mixing [6, 7], level of education [40], and socioeconomic status [39].

Two studies reported seroprevalence rates among HCWs, who are at risk of acquiring varicella as adults and transmitting it to vulnerable patients. In a study in Brazilian neonatal units, 30% of the HCWs lacked a clear history of varicella and ~ 1% (two HCWs) had equivocal immunity based on serology [28]. A study of 89 medical residents in a pediatric hospital in Mexico identified one seronegative individual [41]. The authors recommend that HCWs without a clear positive history of varicella be assessed for antibody levels and if found to be susceptible, vaccinated [28, 41].

### Varicella incidence

Passively reported varicella incidence data underestimate the true burden of varicella, as many patients with varicella do not seek healthcare. Varicella is not a notifiable disease in all countries of LAC, although related hospitalizations and deaths are notifiable. Consequently, the burden of varicella infection and its economic impact on factors such as work absenteeism and decreased income are not routinely captured.

#### Varicella incidence before introduction of universal vaccination

Seven studies from the SLR, and one (reported in Spanish) identified by the authors, provided published data on the incidence of varicella in LAC (see Additional file 3).

Before the introduction of universal vaccination, the annual reported incidence rate ranged from 301 to 437/100,000 population in Costa Rica (data from 2002 to 2006) [22] and from 233 to 381 cases/100,000 (1995–2010) in Mexico [42]. Varicella incidence tended to follow a cyclical pattern, with peaks every few years, as reported in Mexico [42]. A seasonal pattern of infection was also observed, with incidences typically peaking during the winter and spring; this was noted in both tropical (Mexico) and temperate (Uruguay, Argentina, Brazil) countries [42]. The highest incidence of varicella was reported in children aged < 10 years [22, 30, 42].

In Colombia the reported incidence increased over time, from 78 cases/100,000 population in 2008 to 166 cases/100,000 in 2012 [37]. Another epidemiologic study of varicella in Colombia, performed between 2010 and 2014 in the department of Casanare, identified highest rates of infection in urban regions (81.2% of cases), with the 15–44 year age group the most commonly affected; the rate of lethality of cases was 0.1% [51].

**Fig. 5:**
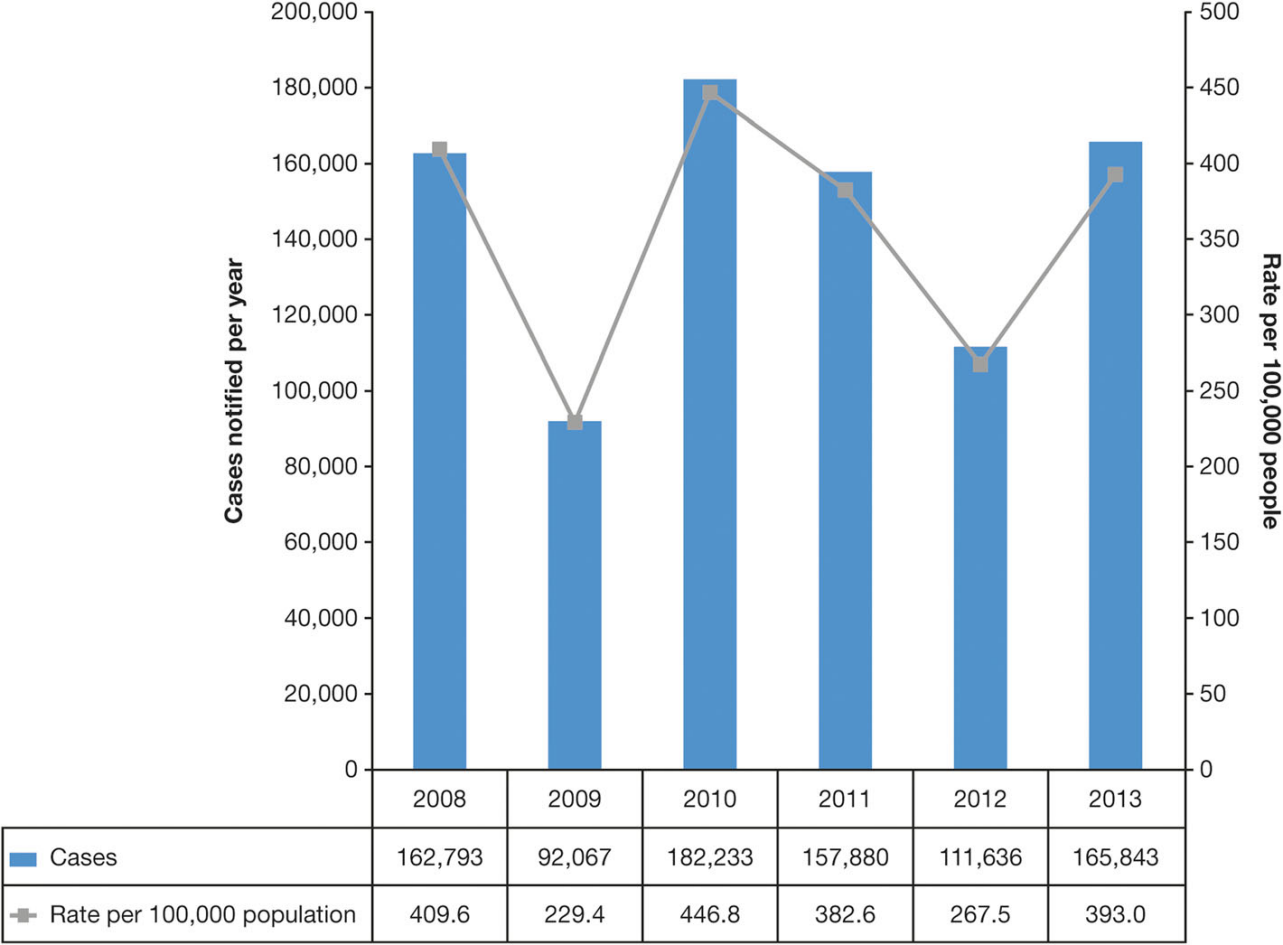
Cyclical pattern of notified cases of varicella in Argentina, 2008 to 2013 [56]

**Fig. 6:**
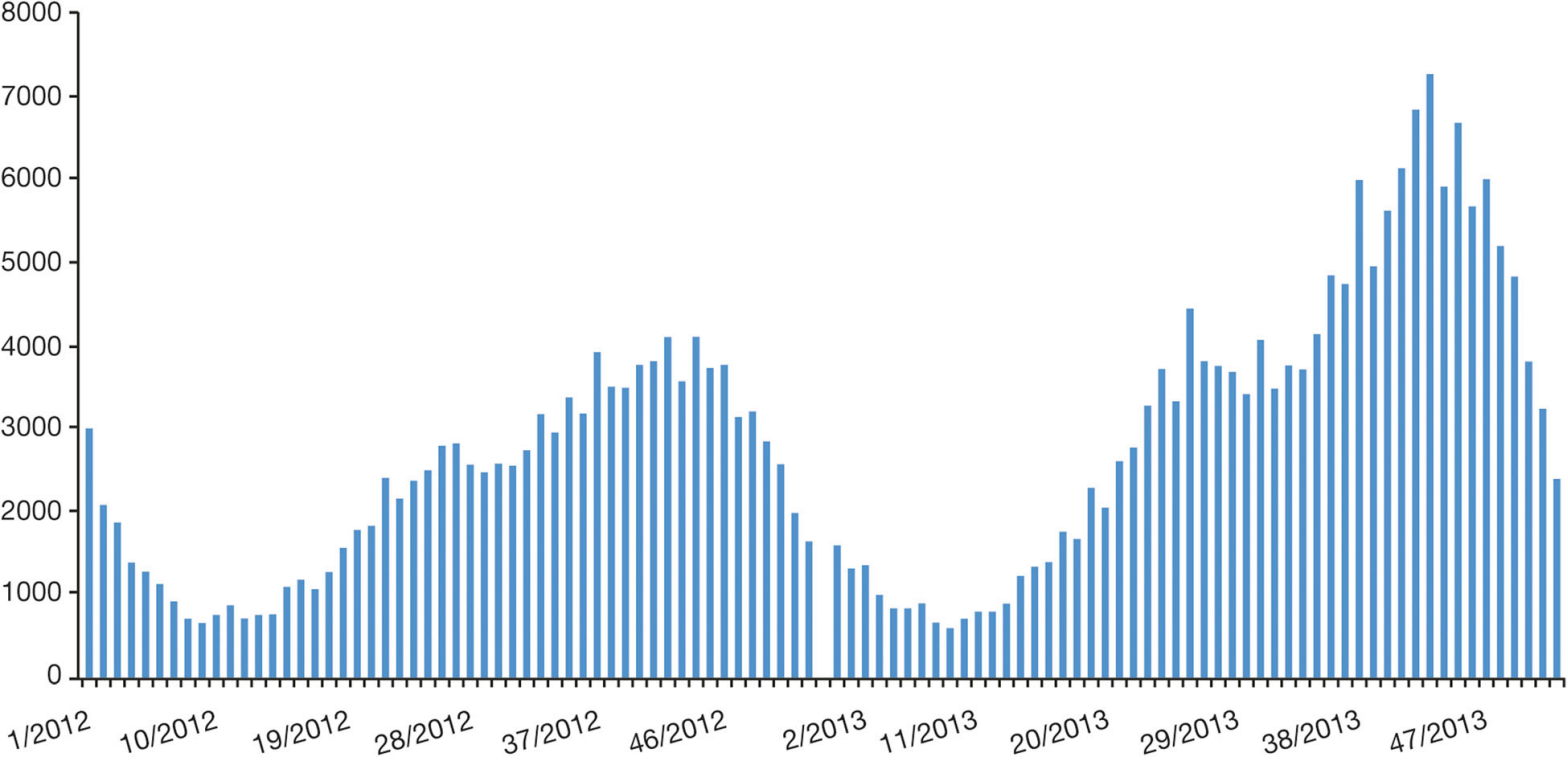
Seasonality of notified cases of varicella in Argentina, 2012 and 2013 [56]

Among other gray literature, SLIPE in 2016 [10, 18, 52] reported incidence rates of 147/100,000 in Venezuela, 148/100,000 in Uruguay, 213/100,000 in Colombia, and 393/100,000 in Argentina. Barbados recorded 1345 reported cases in a population of 270,000 for the 2008–2010 period, and Jamaica recorded 2646 cases of varicella in a population 2.7 million in 2010 [53, 54]. Ministry of Health data in Bolivia reported 120 cases/100,000 population [52]. National statistics for Paraguay supported the trend toward increased incidence of varicella reported previously in the 3 years of 2010–2012 that contributed to the case for introduction of vaccination in 2013 [55]. The aforementioned cyclical pattern and seasonality of notified cases of varicella can be seen in a report of Ministerio de Salud in Argentina [56] (Figs. 5 and 6).

**Fig. 7:**
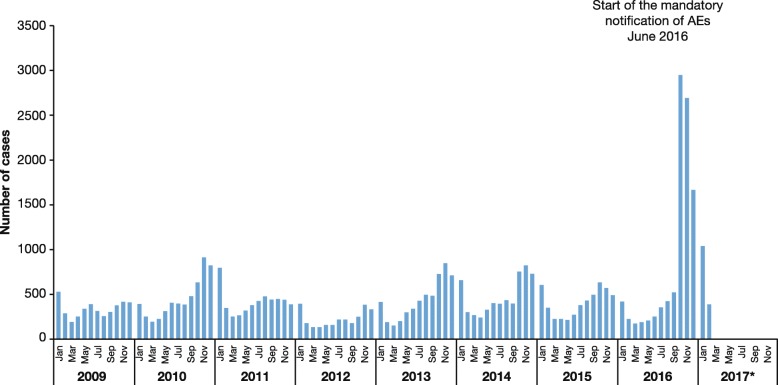
Number of recorded cases of varicella in Peru, 2009–2017. Mandatory reporting was introduced in 2016 [57]

Few countries in LAC enforce mandatory notification. Obligatory notification of varicella cases has an important impact on the number of recorded cases, as demonstrated by a report from the Ministerio de Salud in Peru [57] (Fig. 7).

#### Varicella incidence after introduction of vaccination

A dramatic reduction in the incidence of varicella cases was reported after introduction of varicella vaccination into national immunization programs in Costa Rica and Uruguay, and following the introduction of vaccination in Florianópolis, the state capital of Santa Catarina in Brazil.

**Fig. 8:**
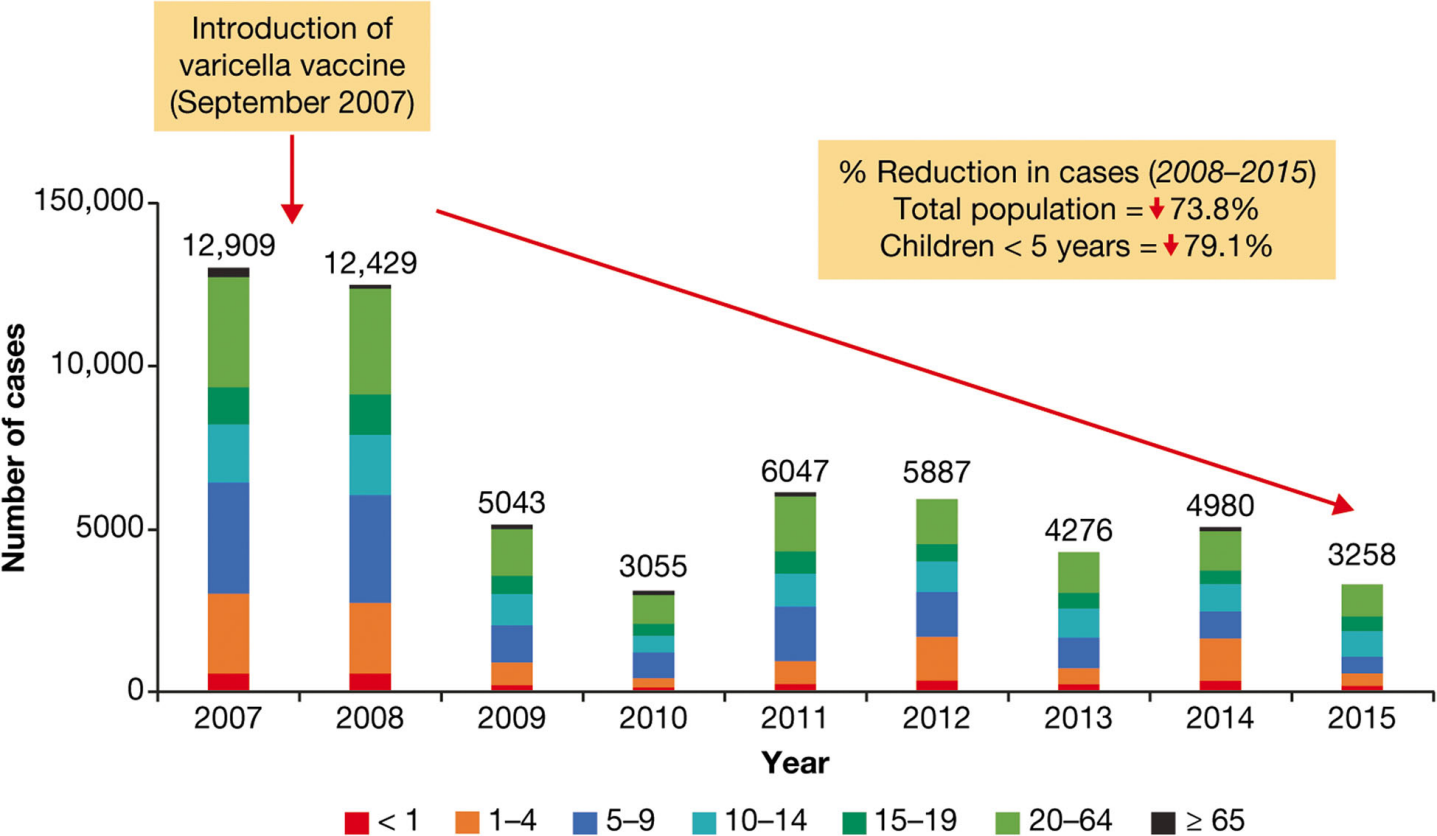
Reported varicella cases by age before and after the introduction of varicella vaccine in Costa Rica [22]. From Avila-Aguero, M. L., R. Ulloa-Gutierrez, K. Camacho-Badilla, A. Soriano-Fallas, R. Arroba-Tijerino and A. Morice-Trejos (2017). “Varicella prevention in Costa Rica: impact of a one-dose schedule universal vaccination.” Expert. Rev. Vaccines 16(3): 229–234 with permission

In Costa Rica, one-dose vaccination was introduced in September 2007 for every child at age 15 months. Between 2008 and 2015, there was a 74% reduction of reported varicella cases to 67 cases/100,000 inhabitants in 2015 (Fig. 8) [22].

In Uruguay in 2005 (6 years after the introduction of a varicella vaccination program), the incidence of ambulatory visits for varicella among children recorded by private insurance organizations was reduced by 87% overall relative to pre-vaccination, and by 80, 97, 81, and 65% in the < 1-, 1–4-, 5–9-, and 10–14-years age groups, respectively [15]. In 2009, the incidence in Uruguay was 20/100,000 [23]. In the Florianópolis, after initiation of varicella vaccination targeting all children < 2 years old in 2002, there was a 75.5% reduction in incidence among the group of children aged 1–4 years; this contrasted with a rising incidence in this age group in the rest of the state, which had not implemented vaccination [30].

Varicella incidence data in São Paulo, Brazil, from 2002 to 2017 show a trend to increasing numbers of cases up to introduction of vaccination in 2013 (25,052), with a fall in cases in subsequent years (2822 in 2017) [58]. A case-control study of children aged 15–35 months, following the national introduction of varicella vaccination in Brazil in 2013 for children at age 15 months, showed the effectiveness of single-dose vaccine to be 86% for disease of any severity and 93% for moderate/severe cases [45]. There was a breakthrough rate of 22%, potentially attributable to vaccine failure, as the cases had been vaccinated only 9 months before, on average; patients with breakthrough varicella had less severe disease than non-breakthrough cases.

### Complications

Five published studies reported data on the complications associated with varicella in LAC (see Additional file 3). Three studies provided data for Brazil [31, 32, 45] and one study each for Argentina [26] and Uruguay [15].

#### Complications pre-vaccination

Among children in Brazil who acquired varicella after starting daycare, 6% experienced complications, including bacterial skin infections (5%), pneumonia (0.5%), sepsis (0.3%), and otitis/sinusitis (0.3%). Children aged < 12 months had a higher risk of complications (14.3%) than those of other ages (5.7%) [31].

Among hospital inpatients aged 1–12 years with varicella in a study in Argentina, 99% had at least one complication, most commonly skin or soft tissue infection, pneumonia, sepsis, cerebellitis, and febrile seizure; by comparison, 28% of outpatients aged 1–12 years with varicella had one or more complications [26]. In a university hospital study in Brazil, 60% of inpatients with varicella had associated complications, most commonly bacterial skin infection (47%) and respiratory (4%), renal (3%), and central nervous system (2%) complications [32].

Miranda-Choque et al. [49] investigated 1073 children (mean age 2.5 years) with complicated varicella infection admitted to a national pediatric hospital in Peru between 2001 and 2011. The most frequent complications were secondary skin and soft issue infections (*n* = 768 cases; 72%). Changes in the number of these complications over time [49] suggest a temporal increase. Thirteen deaths (1.4%) were recorded over this period.

Within the gray literature, varicella disease severity in children was compared between Argentina, Peru, and Mexico (*n* = 230 patients in total) as part of a resource utilization analysis [59]. Patients treated in Mexico had the least severe disease, assessed by the number of lesions per patient, while patients treated in Peru had the most severe disease. Argentina and Mexico had very similar rates of complications, with approximately one in four patients having only one complication.

#### Complications post-vaccination

Vaccinated children with breakthrough varicella experience fewer complications than unvaccinated children, and breakthrough cases tend to be milder. A retrospective study by Quian and colleagues included 294,831 patients in the period from 1997 to 2005 in Uruguay; 7% of children with breakthrough varicella had complications, compared with 12% of unvaccinated children [15].

Canziani et al. [47] investigated cases of varicella outbreaks in Uruguay in 2013, following introduction of varicella vaccination in 1999 in children aged 12 months. Among 151 cases of varicella infection detected in educational centers of one department, 97% were in vaccinated children. There were no serious cases and the frequency of complications was low (4%). Of note, only one third of these cases were reported to the Public Health Ministry.

### Mortality and case fatality

#### Mortality pre-vaccination

Limited data are available from published studies on the mortality associated with varicella (see Additional file 3). Three relevant studies were conducted in Brazil [30, 32, 33, 46] and one study each in Colombia [37] and Costa Rica [22].

**Fig. 9:**
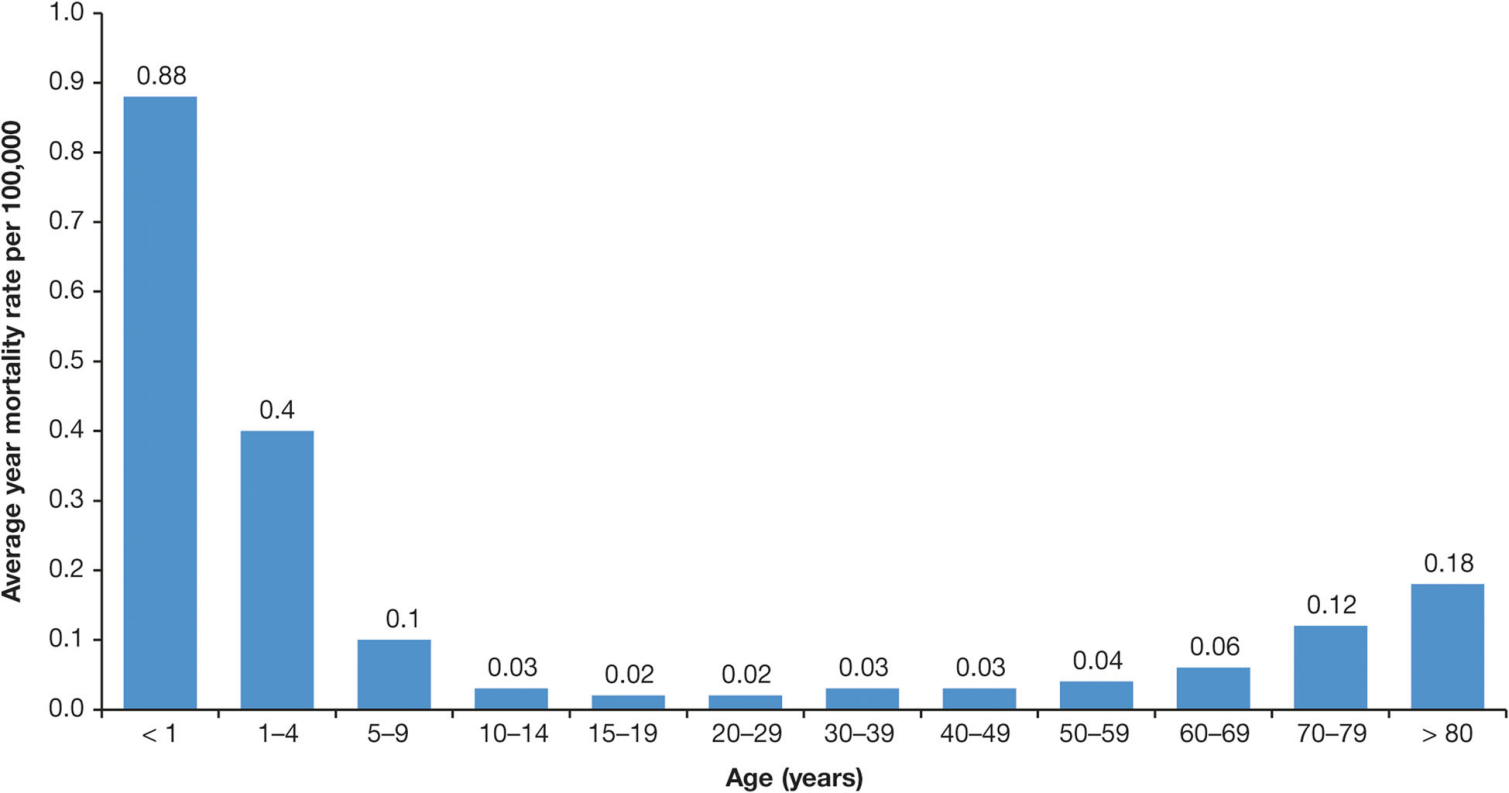
Average annual mortality rates for varicella zoster virus per 100,000 by age group in Brazil, 1996–2011 [33]

Average annual mortality rates for varicella in Brazil between 1996 and 2011 ranged from 0.88 cases/100,000 population aged < 1 year to 0.02 cases/100,000 population aged 15–19 years (Fig. 9) [33]. In total over this period, 2334 varicella-associated deaths were reported; the authors note that this represents almost one death every 2 days.

A study of varicella-related mortality in children aged < 7 years attending municipal daycare centers in the city of São Paulo, Brazil, reported 12 deaths in the period 1996–1999, a rate of 0.240/100,000 [46, 60]. By comparison, the rate of varicella-related death rates for all children in the county was 0.105/100,000 [60].

The fatality rate was 2% among Brazilian inpatients with varicella (*N* = 255) in the period 2004–2005; causes of death included pneumonia-associated encephalitis, hemorrhagic varicella associated with septicemia, and immunosuppression [32].

**Fig. 10:**
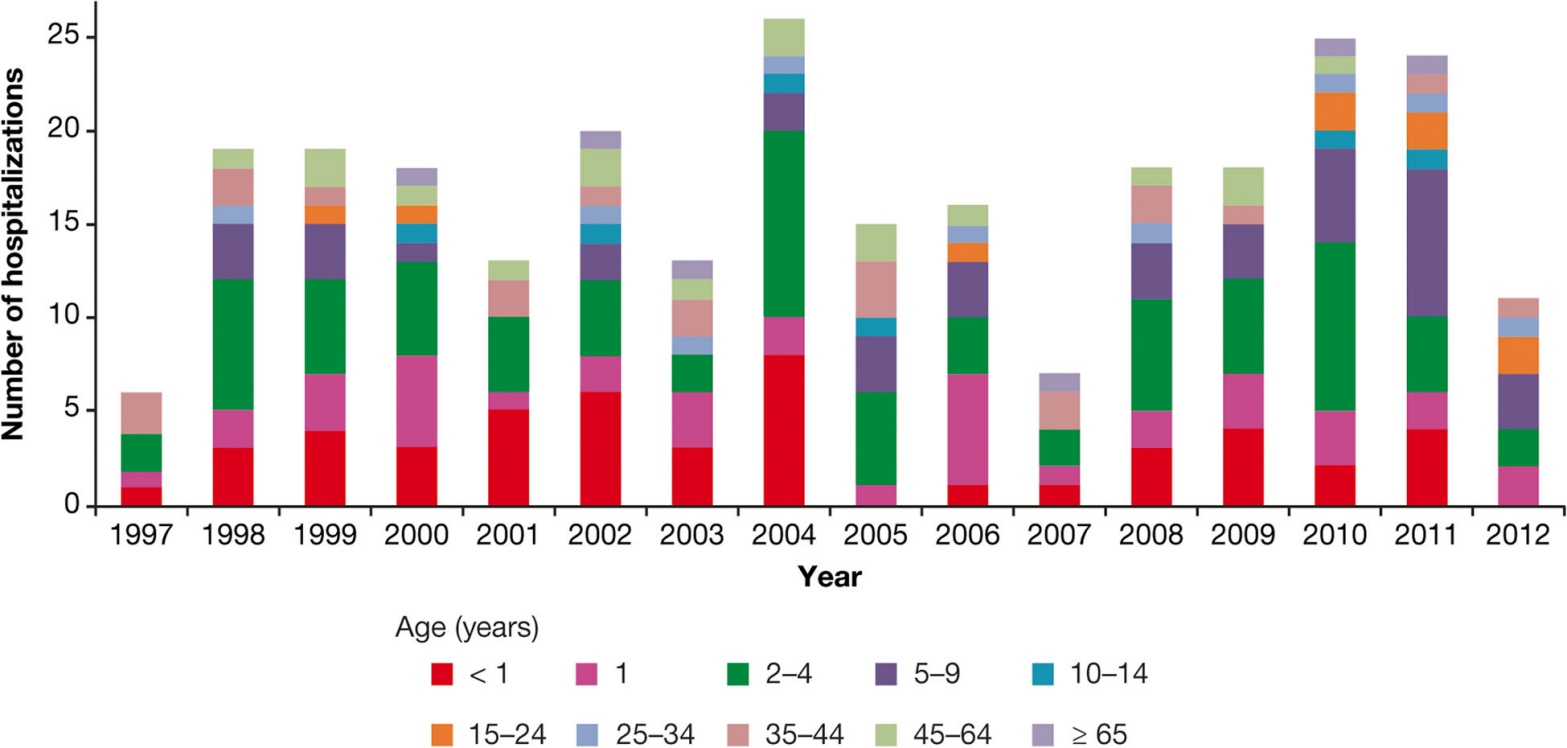
Mortality due to varicella grouped by age in Argentina, 1997 to 2012 [56]

An analysis of varicella cases leading to death by the Ministerio de Salud in Argentina [56] showed that deaths from this cause occurred in all age groups (Fig. 10).

#### Mortality post-vaccination

In Costa Rica, the number of deaths associated with varicella in nationwide surveys was 23 in the pre-vaccination era (2000–2007) and 24 in the post-vaccination era [22], therefore showing no evident differences (2008–2014); however, no information was available on the vaccination status of the deceased patients.

### Economic burden of varicella infection

In total, eight published studies provided evidence for the economic impact of varicella in the LAC region, with data from Argentina [26], Brazil [28, 31–33], Colombia [37], Costa Rica [22], and Uruguay [15].

### Healthcare resource utilization and hospitalization

#### Healthcare resource utilization pre-vaccination

Seven published studies have reported on resource utilization or hospitalization rates in the LAC region (see Additional file 3). Each study provided resource use data in different settings.

#### Hospital-based healthcare

High rates of hospitalization are reported following varicella infection. Case hospitalization rates of approximately 1% were reported in Colombia and Brazil before introduction of universal vaccination [31, 37]. In Brazil, data from the Department of the Unified Health System show that around 62,000 hospitalizations related to VZV occurred in 2008–2009, with the majority of cases occurring in patients < 9 years and peaks in the 1–4-year age group (27%) and with a higher monthly average from September to November [33]. The authors noted that, over a year, this averaged at 34 hospitalizations per day in Brazil.

The average duration of hospitalization for varicella in these studies was approximately 1 week. In Argentina, the average length of stay for children aged 1–12 years during 2009–2014 was 5 days; the total combined direct and indirect cost per varicella case (2015-dated) was US $2947.7 (inpatients) versus US $322.7 (outpatients) (equivalent to US $3109.2 and $339.6, respectively, 2017-dated) [26]. In Brazil, the average reported length of hospital stay was 7.5 (mean) [32] to 8.5 days (median) [31]. Before introduction of UVV in Costa Rica and Uruguay, the mean length of stay was 4.85 days and 3–5 days, respectively [15, 22].

A poster presentation by Castillo et al. [59] describes a retrospective chart review of six public and three private sites in Peru for children aged 1–15 years with a primary diagnosis of varicella. Inpatients (*n* = 78) more frequently had ≥ 50 lesions (98.7%) compared with outpatients (*n* = 101; 44.6%). Total mean costs were Sol. 2634.8 and Sol. 334.1 (2016-dated [US $1690.8 and US $214.4, 2017-dated]) in inpatients and outpatients, respectively, including direct costs of Sol. 1874.8 and Sol. 121.5 (US $1203.1 and US $78.0, 2017-dated), respectively. The total estimated annual population cost associated with varicella in Peruvian children (aged < 15 years) was estimated at Sol. 47,595,390/US $14,102,338 (2016-dated [US $30,541,883, 2017-dated). The authors commented that this substantial economic impact of varicella in Peru is supportive of a routine childhood varicella vaccine plan.

Vazquez et al. [61] reported on the economic burden of varicella in Mexico, based on retrospective chart study of children aged 1–14 years with varicella (*n* = 77 inpatients, *n* = 75 outpatients). Total mean costs per case were Mexican peso (MXN) $113,454.82 (US $5786.20) in inpatients and MXN $4718.57 (US $240.65) in outpatients (2017-dated), with direct costs accounting for MXN $110,019.41 (US $5610.99) and MXN $3888.52 (US $198.31), respectively. Significant amounts of medication were used in the treatment of varicella in both inpatient and outpatient settings. Total estimated annual population costs were MXN $2.4 billion (range, 2.01–3.14 billion) (US $123,500,00.00 [range, $102,394,788.00–$160,055,878.00]). As with the study by Castillo et al. in Peru, the authors concluded that increasing urbanization and school attendance may be contributing to an increase in varicella disease burden.

Finally, a comparison of resource utilization among hospitalized pediatric patients in Argentina, Peru, and Mexico identified substantial healthcare resource use in each country, with wide variations in the direct cost per hospitalized patient across the three countries (Argentina US $2804.60, Mexico US $5610.99, and Peru US $547.80, 2017-dated) [62]. Variation in costs was highly reflective of the cost per day of hospitalization and to the gross national income per capita.

### Treatments and tests

Vazquez et al. [61] analyzed prescription medication use for treating varicella in children aged 1–14 years in Mexico, and reported that the average duration of prescription medications was 18.0 days in inpatients and 7.1 days in outpatients. Overall, 53% of prescription medications were antibiotics. A similar analysis of prescription medication use in Peru reported that 80% were antibiotics [59].

### Healthcare resource utilization post-vaccination

**Fig. 11:**
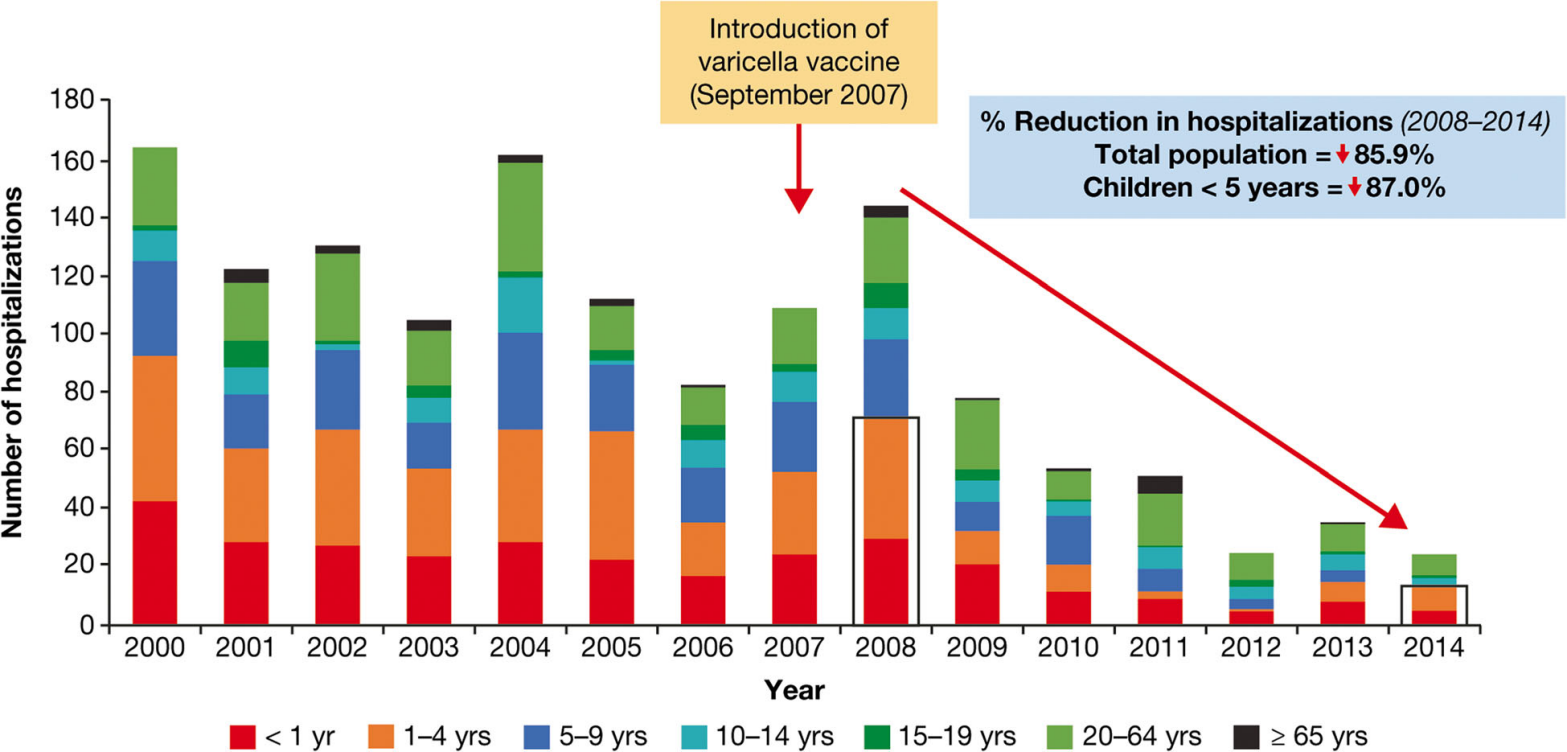
Varicella hospitalizations by age group before and after introduction of varicella vaccine in Costa Rica, 2000 to 2014 [22]. From Avila-Aguero, M. L., R. Ulloa-Gutierrez, K. Camacho-Badilla, A. Soriano-Fallas, R. Arroba-Tijerino and A. Morice-Trejos (2017). “Varicella prevention in Costa Rica: impact of a one-dose schedule universal vaccination.” Expert. Rev. Vaccines 16(3): 229–234 with permission

The introduction of single-dose varicella vaccination programs has led to a dramatic reduction in hospitalization in Costa Rica and Uruguay. In Costa Rica, an 86% reduction in varicella hospitalizations was reported nationwide (87% in children aged < 5 years) for the post- (2008–2014) versus pre- (2000–2007) vaccination era [22]. Hospitalizations for complicated varicella decreased by 98%. In 2008, there were 53 hospitalizations due to varicella complications (pneumonia, meningitis, or encephalitis), which reduced to one in 2014 (Fig. 11).

**Fig. 12:**
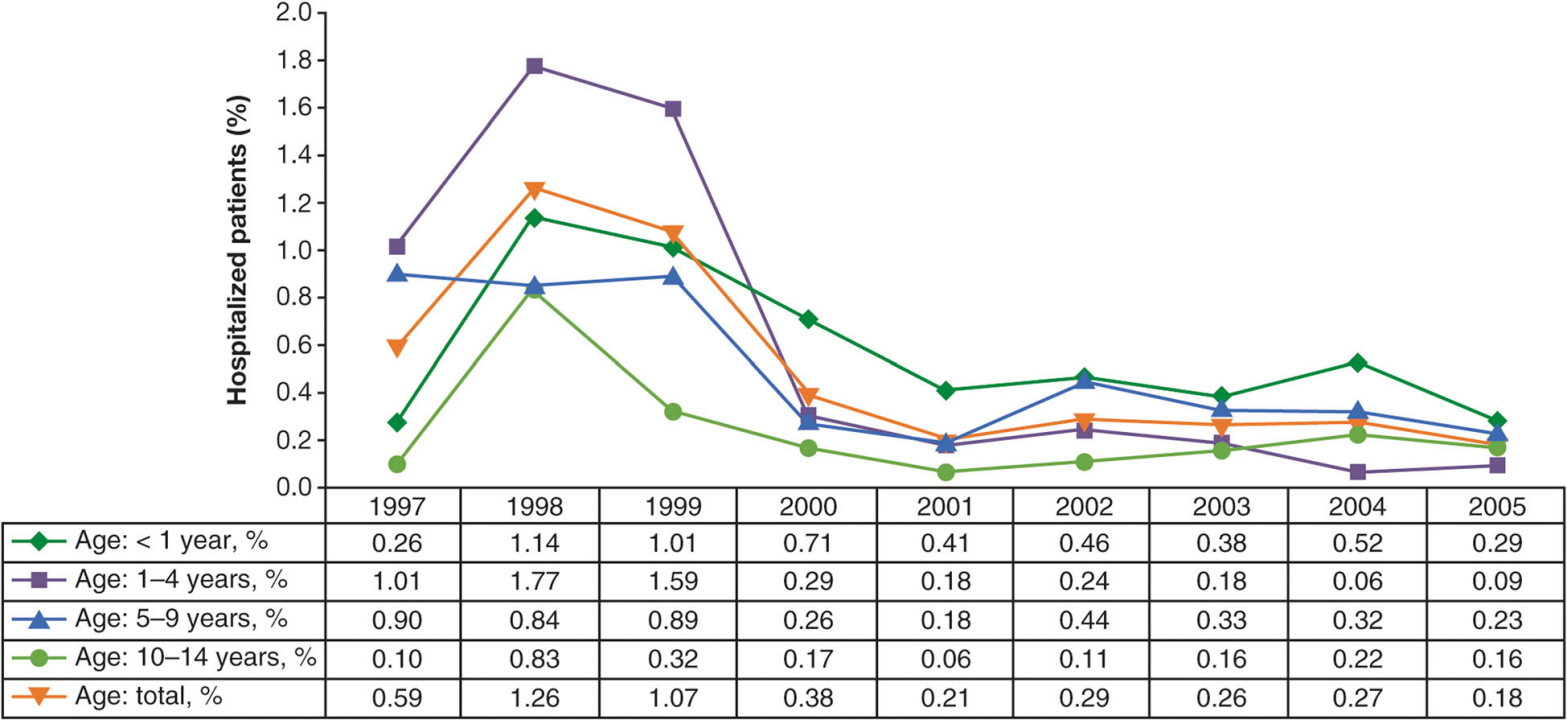
Varicella hospitalizations by age group before and after introduction of varicella vaccine in Uruguay, 1997 to 2005 [15]

In Uruguay, hospitalization rates in children decreased by 81% compared with pre-vaccination years (1997–1999), including decreases of 63, 94, 73, and 62% for < 1-, 1–4-, 5–9-, and 10–14-years age groups, respectively [15] (Fig. 12).

In Puerto Rico, there has been a substantial decrease in the morbidity associated with varicella following the introduction of vaccination – from 11.6 cases/100,000 in 1998 to 2.8 cases/100,000 in 2015 (Departamento de Salud, Puerto Rico, cited by Avila-Aguero et al.) [10].

### Economic burden in absence of vaccination

Limited data are available from four published studies to describe the economic burden of varicella in LAC. The major contributor to direct costs is hospitalization [63, 64]. For example, the cost for the hospitalization period per varicella case in Colombia was estimated at US $151, 2008-dated [equivalent to US $176, 2017-dated] [64]. Other major costs included specialized management (US $53 [US $62]), treatment costs (US $41 [US $48]), laboratory testing costs (US $23 [US $27]), and consultation costs (US $8 [US $9.3]).

From the societal (non-medical) and healthcare system perspective in Brazil, the mean estimated cost per varicella case by age groups of < 1, 1–4, 5–9, and 10–14 years can be seen in Table 3 [63].

**Table 3:** Cost per varicella case in Brazilian Reals (R $), 2004-dated [and equivalent US $, 2017-dated] from societal and healthcare perspectives in Brazil [63]

Age, years	Societal Perspective		Healthcare System Perspective	
	Outpatient Healthcare Facilities	Hospital	Outpatient Healthcare Facilities	Hospital
<1	R $23.34	R $454.32	R $12.51	R $353.59
	(US $29.78)	(US $579.57)	(US $15.96)	(US $451.07)
1–4	R $22.14	R $472	R $11.67	R $370.3
	(US $28.25)	(US $602.13)	(US $14.88)	(US $472.39)
5–9	R $21.04	R $473.18	R $10.91	R $371.82
	(US $26.84)	(US $603.63)	(US $13.91)	(US $474.32)
10–14	R $28.37	R $455.23	R $13.83	R $362.31
	(US $36.19)	(US $580.73)	(US $17.65)	(US $462.19)

### Economic evaluation of vaccination

Three studies provided economic evaluations of vaccination prior to the introduction of universal vaccination programs in Colombia [64, 65] and Brazil [63]. All three studies utilized a decision analysis model, but each study was conducted with a different perspective: societal [63], payer [65], or provider [64]. The time horizon was 30 years in all studies.

The Colombian analysis by Paternina*-*Caicedo et al. [64] predicted that, in an average year, there would be 700,197 varicella cases and 60 deaths in the country in the absence of vaccination, with healthcare costs of around US $88,734,735, 2008-dated [US $101,023,480, 2017-dated] (with discount) over a 30-year period. Vaccination effectiveness was estimated at 85% for the one-dose and 95% for the two-dose vaccine with vaccination coverage of 80%. The cost per life-year gained (2008-dated) of one-dose and two-dose vaccination was US $2519 and US $5728, 2008-dated, respectively [US $3501 and US $6652, 2017-dated] (Table 4). The authors concluded that vaccinating against varicella in Colombia is cost-effective under the assumptions used [64].

**Table 4:** Cost-effectiveness ratio in US $, 2008-dated [equivalent US $, 2017-dated] for one-dose and two-dose varicella vaccine in Colombia [64]

Cost	One-Dose Vaccine Compared With No Vaccine (ACER)	Two-Dose Vaccine Compared With No Vaccine (ACER)	Two-Dose Vaccine Compared With One-Dose Vaccine (ICER)
Cost per avoided consultation	$9 (equivalent to $12.5)	$21 (equivalent to $29.2)	$119 (equivalent to $165.4)
Cost per avoided hospitalization	$4893 (equivalent to $5681.9)	$11,127 (equivalent to $11,5463.7)	$64,110 (equivalent to $89,096.7)
Cost per avoided death	$75,187 (equivalent to $87,309.1)	$170,962 (equivalent to $237,594.0)	$983,977 (equivalent to $1,367,479.3)
Cost per LYG	$2519 (equivalent to $3318.76)	$5728 (equivalent to $6305.69)	$33,002 (equivalent to $43,479.91)
Cost per DALY averted	$1362 (equivalent to $1892.8)	$3097 (equivalent to $4304.1)	$17,844 (equivalent to $24,798.7)

*ACER* average cost-effectiveness ratio; *DALY* disability adjusted life-years; *ICER* incremental cost-effectiveness ratio; *LYG* life-years gained

In a second study in the Colombian setting, De La Hoz et al. [65] concluded that the varicella cost with vaccination was lower than the varicella cost without vaccination (US $35 million vs. US $88 million in 2008 [equivalent to US $40.6 million vs. US $102.2 million in 2017]), resulting in an incremental cost-utility ratio of US $2527 per life-year gained (LYG) in 2008 [equivalent to US $2934.4 per LYG in 2017] [65].

The cost-effectiveness analysis published in 2008 by Valentim et al. [63] compared two strategies for varicella vaccination in Brazil: universal vaccination in 12-month-old children and targeted vaccination of individuals at high risk for severe disease. Assuming a single-dose schedule with vaccine efficacy of 85% and coverage of 80%, a universal childhood vaccination program could prevent 74,422,058 varicella cases and 2905 deaths over 30 years, at a cost of R $3,178,396,110 (US $1,887,495, 2017-dated) with a saving of R $660,076,410 (US $384,573,432.9, 2017-dated) to society and R $365,602,305 (US $213,007,057.46, 2017-dated) to the healthcare system [63]. The universal program is concluded to be cost-effective, with a cost per life-year saved of R $14,749 and R $16,582 (US $8593 and $9661, respectively, 2017-dated) from societal and healthcare system perspectives, respectively [63] (Table 5). However, a potential limitation of this analysis is that the varicella hospitalization rate in age groups above 40 or 50 years may contain some cases of zoster as, in Brazil, varicella zoster virus-associated hospitalizations are reported according to an uncoded morbidity list (including both varicella and zoster diseases).

**Table 5:** Cost incurred in Brazilian Reals (R $), 2004-dated [and equivalent US $, 2017-dated] in current strategy and universal strategy for vaccination in Brazil [63]

Cost	Society		Healthcare System	
	Current Strategy (Targeted Vaccination)	Universal Vaccination	Current Strategy	Universal Vaccination
Varicella treatment total cost (direct + indirect)	R $821,368,711 (US $478,551,061)	R $161,292,301 (US $95,782,241)	R $432,378,310 (US $251,915,095)	R $66,776,005 (US $39,652,189)
Total treatment cost avoided	–	R $660,076,410 (US $384,573,433)	–	R $365,602,305 (US $213,007,057)
Vaccination program cost	R $147,739,823 (US $86,077,147)	R $3,178,396,110 (US $1,887,471,936)	R $147,739,823 (US $86,077,147)	R $3,178,396,110 (US $1,887,360,012)

The gray literature included a business impact analysis of introducing a one-dose varicella vaccination program for 13-month-old children in Mexico [66], based on estimates for population size (121 million), assumed vaccination coverage, vaccine acquisition (MXN $285.72 [US $14.69], both 2017-dated) and administration (MXN $25.09 [US $1.29]) costs, and outpatient and hospitalization costs. The projected reduction in number of varicella cases from vaccination was estimated to result in substantial cost savings over a wide range of vaccine coverage scenarios. For low coverage (10%), reductions in annual total direct medical costs ranged from MXN $91 million [US $4.68 million] in year 1 to MXN $397.5 million [US $20.42 million] in year 10. For high coverage (99%), cost savings ranged from MXN $629.9 million [US $32.37 million] in year 1 to MXN $4.3 billion [US $0.23 billion] in year 10.

Cost-effectiveness analyses in Peru compared four vaccination strategies (1 dose at age 12 months, 90% coverage [base case]; one dose at 18 months, 60% coverage; two doses, the first at 12 months, 90% coverage and the second at 18 months, 60% coverage; and two doses, the first at 12 months, 90% coverage and the second at 4 years, 50% coverage) [67]. Estimates based on population size, costs of vaccine and of treatment, and health impact identified the first strategy as the most cost-effective (Sol. 34.11527 per person, 2016-dated [US $21.89241, 2017-dated]). The health benefits from two-dose strategies would likely be significantly higher if the second-dose coverage was assumed to be higher.

A similar analysis in Chile compared five vaccination strategies (one dose at 12 months, 90% coverage (base case); one dose at 18 months, 85% coverage; two doses, 12 months, 90% coverage and 18 months, 85% coverage; two doses, 12 months, 90% coverage and 6 years, 85% coverage; and two doses, 18 months, 85% coverage and 6 years, 85% coverage) versus no vaccination [68]. All vaccination strategies were projected to rapidly reduce varicella incidence by at least 95% within 5 years. The most effective strategy—with lowest overall varicella incidence, lowest breakthrough varicella incidence, and lowest age-specific varicella incidence in all age groups—was achieved by the first dose at 12 months and the second dose at 18 months.

Argentina introduced first-dose UVV in 2015 for children aged 15 months, intending to eventually move to a two-dose schedule within 5 years. Giglio et al. [69] evaluated the cost, health impact, and cost-effectiveness of different two-dose vaccination strategies (monovalent vs. MMRV; short or long interval), considering both societal (direct and indirect costs) and payer (direct costs only) perspectives. The long interval schedule with monovalent vaccines was both the least expensive and the most cost-effective strategy.

The cost-effectiveness of vaccination strategies in Mexico was investigated in a dynamic transmission model [70]. Five vaccination strategies were compared with the current status quo of no vaccination (1 dose; 1 dose + catchup second dose; 1 dose + campaign [to vaccinate 95% of 14-month-olds with 95% coverage]; 2 doses; and 2 doses + campaign). All five scenarios were cost saving. The one-dose + campaign vaccination strategy was the most cost saving, having a saving of MXN $722 per person (US $37.1, both 2017-dated) over 25 years. The two-dose + campaign vaccination strategy had the strongest health impact, with an incremental cost-effectiveness ratio of MXN $3.815 × 10^8^ (US $0.196 × 10^8^)/QALY (2017-dated) when compared with the primary plus campaign vaccination strategy over 25 years.

## Discussion

This review highlights the substantial clinical and economic burden of varicella in the LAC region in the absence of UVV, supplementing previous data published in 2012 from Bardach et al. [11], with significant new data on seroprevalence and economic burden, implementation of varicella vaccination programs across the region, and evaluation of the implementation of UVV. Seroprevalence data across LAC show high rates in childhood, with continued increases in rates into adulthood. Varicella incidence rates are less reliable due to under-reporting and the lack of mandatory notification of all cases, although they show consistent trends of seasonal variation with annual peaks and troughs across the region where such data are available. Increased urbanization, levels of crowding, and school-age mixing are contributing to an increase in varicella infection incidence over time. When complications occur, they can be serious, leading to hospitalization and, in some cases, to death. As de Martino Mota et al. [33] noted in the Brazilian setting, varicella cannot be considered a benign disease. The cost estimates reported in this paper, particularly in relation to hospitalization in the absence of vaccination, are likely to be greatly underestimated, which impacts the calculations of the cost-effectiveness of vaccination.

The introduction of mandatory UVV in countries in LAC—including Costa Rica and Uruguay—has had a substantial beneficial effect on reducing varicella incidence. It is expected that the varicella burden will further decrease in these countries as more cohorts of children are vaccinated and herd immunity increases. The recent expansion of UVV in other countries in the region is very encouraging, and more than half the population is now living in countries with a universal vaccination program [52]. Many countries in LAC, including populous countries such as Mexico and Venezuela, do not universally vaccinate for varicella, and only a few countries use the two-dose schedule as recommended by SLIPE to reduce the number of cases and outbreaks, with the potential to eliminate and eradicate the disease [10, 18]. In this regard, the Revolving Fund of the PAHO, which is a cooperative mechanism for the joint procurement of vaccines, syringes, and related supplies for participating member states, is strengthening these countries’ capabilities regarding immunization [71].

Modeling studies based on the available data show that universal vaccination (one- or two-dose) with highly effective varicella vaccines provides societal and healthcare cost reductions compared with no vaccination in each country analyzed. While there remains a need for additional local data, current evidence in LAC, as described in this review, provides an impelling rationale for the wider implementation of vaccination in this region.

There are a number of limitations in our review relating to the interpretation of the burden of varicella in LAC: the paucity of published data in LAC, a high likelihood that the data available is an underestimate of the burden and costs of varicella, and the fact that many countries in LAC are not represented in the manuscript at all, as no data have been collected or analyzed.

## Conclusions

Varicella continues to pose a substantial burden in the LAC region. Thus, UVV provides important reductions in the number and severity of varicella cases. Countries in the region that do not currently have routine national childhood immunization should consider the introduction of UVV within the national immunization program as stated in World Health Organization and SLIPE recommendations. The challenge for countries that have already implemented UVV is to maintain high rates of coverage, monitor for vaccine effectiveness, disease outbreaks, and changing patterns of varicella and zoster, and, where relevant, consider inclusion of a second dose or catch-up campaign within high-risk groups.

## Additional files

Additional file 1: Protocol eligibility criteria. The protocol that defines the predefined objectives and eligibility criteria for inclusion of studies. (DOCX 21 kb)

Additional file 2: Search strategy in Embase® and MEDLINE® using Embase.com platform. Search criteria for the identification of relevant studies on Embase® and MEDLINE®. (DOCX 39 kb)

Additional file 3: Summary of published studies from Latin America and the Caribbean. Listing of the main characteristics and outcomes from the selected published studies. (DOCX 39 kb)

## Abbreviations

ACER: Average cost-effectiveness ratio
CI: Confidence interval
DALY: Disability-adjusted life-years
HCW: Healthcare worker
ICER: Incremental cost-effectiveness ratio
LAC: Latin America and the Caribbean
LILACS: Latin American and Caribbean Health Sciences Information database
LYG: Life-years gained
MMRV: Measles, mumps, rubella, varicella
PAHO: Pan American Health Organization
PRISMA: Preferred Reporting Items for Systematic Reviews and Meta-Analysis
SLIPE: Latin American Society of Pediatric Infectious Diseases (Sociedad Latinoamericana de Infectologia Pediátrica)
SLR: Systematic literature review
VZV: Varicella-zoster virus
