# Supplementary material for: Burden of varicella in Latin America and the Caribbean: findings from a systematic literature review
Source: BMC Public Health. 2019 May 8;19:528. doi: 10.1186/s12889-019-6795-0 (PMC6507223; doi:10.1186/s12889-019-6795-0)
Supplement: Supplementary file 1 — Protocol eligibility criteria. The protocol that defines the predefined objectives and eligibility criteria for inclusion of studies. (DOCX 21 kb) [file 12889_2019_6795_MOESM1_ESM.docx]

**Additional file 1** Protocol eligibility criteria

| Objectives and Research Questions | |
| --- | --- |
| Primary objective | Epidemiology review   - To conduct an epidemiological review to assess the incidence/prevalence, seroprevalence, and case fatality rates of varicella (chickenpox) infection   Economic burden review   - To examine the economic burden of varicella, including healthcare resource utilization   Vaccination coverage program objective   - To provide data on current status of vaccination programs, including coverage rates and economic evaluations of these programs |
| Studies to Include | |
| Patient population | - Age: Individuals 12 months of age or older; studies with infants aged < 12 months will be categorized separately - Sex: Any - Race: Any - Disease: Patients with primary and/or breakthrough varicella (chickenpox) infection |
| Study design | The main study designs to be included are listed below:  Epidemiological burden   - Epidemiological studies, including descriptive studies and ecological studies - Cohort studies, including historical cohort studies and nested case-control studies - Case-control studies - Cross-sectional studies - Registry/database studies - Seroprevalence studies - Routine surveillance reports   Economic burden   - Cost of illness studies - Resource use studies - Cost analyses - Economic evaluations for vaccination programs |
| Language | - English language (Studies with non-English full-text will be identified and list to be shared for consideration for inclusion) |
| Databases | Major biomedical databases   - Embase^®^ and MEDLINE^®^ (using Embase.com platform)   Regional databases   - Index Medicus for the WHO Eastern Mediterranean Region (IMEMR) - Latin American and Caribbean Health Sciences Information (LILACS) database produced by BIREME, the Latin American and Caribbean Center on Health Sciences Inform - Health Literature, Library and Information Services (HELLIS), which also contains Index Medicus for the South-East Asia Region (IMSEAR) |
| Additional data sources | - Bibliographic reference lists of the included studies - Country specific website searching and Internet searching to address any data gaps - Institute for Health Metrics and Evaluation (IHME) website |
| Region/country focus | - Latin America, Middle East, Eastern Europe, and Asia-Pacific |
| Information to Extract | |
| Extraction parameters (list of outcomes to be extracted will be agreed upon prior to data extraction stage)^a^ | Extraction parameters will be finalized after discussion, prior to the data extraction stage. Data extraction would broadly cover the following parameters:   - Study characteristics (setting, country, data source, inclusion/exclusion etc) - Patient characteristics (age, sex, comorbid diseases, baseline disease characteristics, weight, etc) - Economic parameters (perspective, direct and indirect cost, ICER, DALY, QALY, resource use) - Epidemiological parameters (incidence, prevalence, case fatality rates, hospitalization rates) - Vaccination program (coverage rates, costs, recommended doses, recommended target population, etc) |
