# Supplementary material for: Burden of varicella in Latin America and the Caribbean: findings from a systematic literature review
Source: BMC Public Health. 2019 May 8;19:528. doi: 10.1186/s12889-019-6795-0 (PMC6507223; doi:10.1186/s12889-019-6795-0)
Supplement: Supplementary file 2 — Search strategy in Embase® and MEDLINE® using Embase.com platform. Search criteria for the identification of relevant studies on Embase® and MEDLINE®. (DOCX 39 kb) [file 12889_2019_6795_MOESM2_ESM.docx]

**Additional file 2** Search strategy in Embase^®^ and MEDLINE^®^ using Embase.com platform

| No. | Query | Description | Results |
| --- | --- | --- | --- |
| #1 | ‘chickenpox’/exp OR varicella:ab,ti OR ‘chicken pox’:ab,ti OR ‘chickenpox’:ab,ti | Disease facet | [20,475](http://www.embase.com/) |
| #2 | ‘epidemiology’/exp OR epidemi*:ab,ti OR inciden*:ab,ti OR prevalence:ab,ti | Epidemiology studies facet | [3,251,887](http://www.embase.com/) |
| #3 | #1 AND #2 | Combining disease and epidemiology facets | [4778](http://www.embase.com/) |
| #4 | #3 AND ([conference review]/lim OR [editorial]/lim OR [letter]/lim OR [note]/lim OR [review]/lim) | Excluding reviews, letters, commentary, and searches | 1177 |
| #5 | #3 NOT #4 |  | 3601 |
| #6 | #5 AND [2012-2016]/py | Studies for all the key regions of interest will be included post-2012: Latin America, Middle East, Eastern Europe, and Asia-Pacific | 1046 |
| #7 | #5 AND [<1966-2011]/py | Excluding studies prior to 2012 in countries already covered in previous review | 2549 |
| #8 | argentina OR australia OR brazil OR canada OR chile OR china OR india OR israel OR japan OR korea OR malaysia OR mexico OR ‘new zealand’ OR philippines OR russia OR ‘saudi arabia’ OR ‘south africa’ OR taiwan OR thailand OR vietnam OR ‘czech republic’ OR czechoslovakia OR england OR france OR hungary OR ireland OR poland OR scotland OR ‘slovak republic’ OR slovenia OR turkey OR uk OR ‘united kingdom’ OR wales |  | [15,235,019](http://www.embase.com/) |
| #9 | #7 NOT #8 |  | 1137 |
| #10 | #6 OR #9 | Final epidemiology review number | 2183 |
| #11 | ‘economics’/de OR ‘economic aspect’/de OR ‘cost’/de OR ‘health care cost’/de OR ‘drug cost’/de OR ‘hospital cost’/de OR ‘health economics’/de OR ‘pharmacoeconomics’/de OR ‘economic evaluation’/exp OR ‘socioeconomics’/de OR ‘health care financing’/de OR ‘low cost’ OR ‘high cost’ OR health*care NEXT/1 cost* OR ‘health care’ NEXT/1 cost* OR cost NEXT/1 estimate* OR ‘fee’/exp OR ‘budget’/exp OR ‘cost variable’ OR unit NEXT/1 cost* OR economic*:ab,ti OR pharmacoeconomic*:ab,ti OR ‘hospital finance’/de OR ‘financial management’/de OR price*:ab,ti OR pricing:ab,ti OR cost* NEAR/3 (treat* OR therap*) OR ‘cost-effectiveness’ OR ‘cost-utility’ OR ‘cost utility’ OR ‘cost benefit’ OR ‘cost minimisation’ OR ‘cost minimization’ OR ‘budget impact’ OR ‘cost consequence’ OR ‘health care utilization’/de OR health*care NEXT/1 (utilisation OR utilization) OR ‘health care’ NEXT/1 (utilisation OR utilization) OR resource NEXT/1 (utilisation OR utilization OR use) OR cost* | Economic burden facet | 1,418,584 |
| #12 | #1 AND #11 | Combining disease and economic facets | 1391 |
| #13 | #12 AND ([conference review]/lim OR [editorial]/lim OR [letter]/lim OR [note]/lim OR [review]/lim) | Excluding reviews, letters, commentary, and searches | 561 |
| #14 | #12 NOT #13 |  | 830 |
| #15 | #14 AND [2012-2016]/py | Studies for all the key regions of interest will be included post-2012: Latin America, Middle East, Eastern Europe, and Asia-Pacific | 223 |
| #16 | #14 AND [<1966-2011]/py | Excluding studies prior to 2012 in countries already covered in previous review | 606 |
| #17 | argentina OR australia OR brazil OR canada OR chile OR china OR india OR israel OR japan OR korea OR malaysia OR mexico OR ‘new zealand’ OR philippines OR russia OR ‘saudi arabia’ OR ‘south africa’ OR taiwan OR thailand OR vietnam OR ‘czech republic’ OR england OR france OR hungary OR ireland OR poland OR scotland OR ‘slovak republic’ OR slovenia OR turkey OR uk OR ‘united kingdom’ OR wales |  | 15,141,096 |
| #18 | #16 NOT #17 |  | 275 |
| #19 | #15 OR #18 | Final economic review number | 498 |
| #20 | ‘vaccination’/exp OR ‘immunization’/exp OR immunisation:ab,ti OR immunization:ab,ti | Vaccination facet | 267,250 |
| #21 | #1 AND #20 | Combining disease and vaccination facets | 5137 |
| #22 | #21 AND ([conference review]/lim OR [editorial]/lim OR [letter]/lim OR [note]/lim OR [review]/lim) | Excluding reviews, letters, commentary, and searches will include all countries 2012 onward | 1853 |
| #23 | #21 NOT #22 |  | 3284 |
| #24 | #23 AND [2012-2016]/py | Studies for all the key regions of interest will be included post-2012: Latin America, Middle East, Eastern Europe, and Asia-Pacific | 932 |
| #25 | #23 AND [<1966-2011]/py | Excluding studies prior to 2012 in countries already covered in previous review | 2345 |
| #26 | argentina OR australia OR brazil OR canada OR chile OR china OR india OR israel OR japan OR korea OR malaysia OR mexico OR ‘new zealand’ OR philippines OR russia OR ‘saudi arabia’ OR ‘south africa’ OR taiwan OR thailand OR vietnam OR ‘czech republic’ OR Czechoslovakia OR england OR france OR hungary OR ireland OR poland OR scotland OR ‘slovak republic’ OR slovenia OR turkey OR uk OR ‘united kingdom’ OR wales |  | 15,141,096 |
| #27 | #25 NOT #26 |  | 1196 |
| #28 | #27 OR #24 | Final number for vaccination objective | 2128 |
