# Supplementary material for: Burden of varicella in Latin America and the Caribbean: findings from a systematic literature review
Source: BMC Public Health. 2019 May 8;19:528. doi: 10.1186/s12889-019-6795-0 (PMC6507223; doi:10.1186/s12889-019-6795-0)
Supplement: Supplementary file 3 — Summary of published studies from Latin America and the Caribbean. Listing of the main characteristics and outcomes from the selected published studies. (DOCX 39 kb) [file 12889_2019_6795_MOESM3_ESM.docx]

Additional file 3 Summary of published studies from Latin America and the Caribbean

| Source | Country | Location | Study Design | Data Source | Timeframe | Setting | Population | Sample size | Outcomes | | Main findings |
| --- | --- | --- | --- | --- | --- | --- | --- | --- | --- | --- | --- |
| Alvarez y Munoz *et al.* 1999 | Mexico | All states, including all geographic zones | Prospective | Questionnaire and serological survey | 1987–1988 | General population | Both sexes, aged 1–90 years | 3737 | SP | | - Overall SP 87.6% - Susceptibility to infection decreased with   - Age (susceptibility: 17.3%, age 1-4 years to 5.2%, 25–29 years)   - higher educational level (susceptibility: 15.3%, illiterate to 7.5%, college educated) |
| Andrade *et al.* 2018 | Brazil | 2 city municipalities | Case-control | Immunization cards | 2013–2015 | Healthcare units | Both sexes, aged 15-32 months | 469 | Vaccination rates | | - Immunization rates lower in suspected VZ cases versus controls (16.8% vs. 45.4%) - After adjusting for confounders, VZV vaccine effectiveness was 78.6% (95% CI: 61.1, 88.2) for any varicella severity and 86.5% (95% CI: 70.2, 94.1) for moderate/severe cases |
| Avila-Agüero *et al.* 2017 | Costa Rica | National | Retrospective | National surveillance databases | 2000–2015 | Reported varicella cases, hospitaliza-tions | Both sexes, all ages | NR | Vaccination rates, varicella cases, hospitalizations | | - Varicella vaccine coverage increased from 76% in 2008 to 95% in 2015 in children aged 16 months - During this period, 73.8% reduction of reported cases and 85.9% reduction of hospitalizations in general population   Varicella incidence pre-vaccination program:   - Annual incidence rate between 301 and 437/100,000 population (data from 2002–2006) - 30,000 cases annually; incidence rate 600–800/ 100,000; > 80% cases in children < 14 years, with highest incidence in 5–9 year olds - In the 10 years pre-vaccination, 872 hospitalizations, of which 631 (72.4%) were in children  aged < 12 years - Most complications requiring hospitalization were skin and soft tissue infections, encephalitis, gastrointestinal tract disorders, and pneumonia   Post introduction of vaccination:   - Incidence reduced in 2008–2015 by 79.1% among children aged < 5 years and 73.8% in all age groups. Incidence decreased from 288.0/100,000 in 2007 to 67.4 in 2015 - Complications in hospitalized patients decreased 98.1%, from *n* = 53 in 2008 to *n* = 1 in 2014 |
| Bartoloni *et al.* 2002 | Bolivia | 8 rural communities in 1 region | Cross-sectional | Serological survey | 1997 | Community | All, aged 1–85 years | 587 | SP | | - Overall SP 80% - SP higher in females (NS) - Increase in SP with age: 1–4 y 21.2%; 5–9 y 56.9%; 10–14 y 83.7%, > 45 y 98.2% |
| Canziani *et al.* 2015  In Spanish | Uruguay | 1 department | Retrospective | Local databases | 2013 | Educational center | Children aged < 15 years | 151 |  | | - 97% of varicella cases occurred in vaccinated children - Greater number of lesions was associated with greater age and greater persistence of fever - There were no serious cases and the frequency of complications was low (4%) - Only one third of the cases were reported to the Public Health Ministry |
| Castillo & Del-Aguila, 2016 | Peru | Nationwide | Retrospective | Hospital records | 2013 | General population | Both sexes, all ages | 217 | Morbidity, cost utilization | | - Most common complications were: skin and soft tissue over infections (72.4%), central nervous system (13.4%) and respiratory 11.1%. 2 patients died (1%) - The estimated total direct cost for 217 inpatients was  Sol. 538,381.48 - Hospitalization costs represented 64% of the total - The average cost per patient was: Sol. 2481.02 |
| Conde-Glez *et al.* 2013 | Mexico | 1 city, 3 regions | Retrospective | National health survey | 2005–2006 | General population | Both sexes, aged 1–70 years | 3658 | SP | | - Overall SP 85.8% - SP increased with:   - Age (63%, children 1–4 years, 76%, children 5–9 years, 94% adults ≥ 20 years)   - Higher socioeconomic status (97.1%, high; 83.0%, low)   - urban vs. rural populations (91.1%, city; 80.1%–89.5%, rural) |
| Dayan *et al.* 2004 | Argentina | 4 cities;  1 rural area | Cross-sectional | Questionnaire and serological survey | 2002 | Public prenatal healthcare facilities | Women, aged  15–49 years | 2807 | SP | | - - Overall SP 98.5% (95% CI: 98.0, 98.9)   - SP increased with:   - Age (range, 97.6%, 15–19 years; 100%, 35–49 years)   - Crowding (range, 98.0%,  ≤ 2 persons/bedroom; 99.3%, > 2 persons)   - Length of residence (range, 97.2%, < 5 years in same place; 99.0%, ≥ 5 years in same place)   - Positive history of varicella   - PPV 99.4%; NPV 2.5% |
| De La Hoz *et al.* 2011 | Colombia | Nationwide | Cost-effectiveness (30-year time horizon) | National immunization program | 2008 | General population | Both sexes, all ages | National | Cost | | - Vaccination would avoid 9,415,444 consultations, 17,576 hospitalizations, and 1144 varicella deaths - Cost per life-year gained was US $2527 - Varicella costs without vaccination would be US $88 million; with vaccination,  US $35 million |
| de Martino Mota & Carvalho-Costa, 2016 | Brazil | Nationwide | Epidemiologi-cal surveillance | National database | 1996–2013 | Uniﬁed Health System Informatics Department | Both sexes, all ages | National | Varicella-associated mortality, hospitalizations | | - Average annual mortality rates for varicella from 1996 to 2011 were 0.88/100,000 in infants under 1 year and 0.40/100,000 in children aged 1–4 years, decreasing to 0.02 cases/100,000 in those aged 15–19 years - The total number of hospitalizations associated with VZV from 2008 to 2013 was 62,246 - 19.3% (*n* = 450) deaths were in infants < 1 year, 36% (*n* = 840) in children 1–4 years, 11.7% (*n* = 273) in children 5–9 years, and 33% (*n* = 771) in patients  > 9 years - Average annual mortality rates from 1996 to 2011: 0.88/100,000 in infants < 1 year; 0.40/100,000 in children 1–4 years |
| Dinelli *et al.* 2009 | Brazil | 1 city | Retrospective | Questionnaire | 2006–2007 | University hospital | Healthcare workers | 187 | Reported history of disease and vaccination | - 82.9% reported history of varicella or previous vaccination (92.4% physicians, 84.6% nurses, 56.7% housekeepers) | |
| dos Anjos *et al.* 2009 | Brazil | 1 city, 1 metropolitan area | Retrospective | Chart review | 2004–2005 | University hospital | Inpatients admitted for varicella, aged < 1 year to 15+ years | 255 | Complications, cost-effectiveness analysis | | - 60.4% complication rate - Complication type: Secondary skin bacterium infection (46.7%), respiratory (4.3%), renal (3.1%), other (septicemia, pinkeye, arthritis) (3.1%), nervous system (2.3%), hematology (0.7%) - 5 of 255 (2%) deaths (encephalitis associated with pneumonia; pneumonia; hemorrhagic chickenpox associated with septicemia; septicemia; and immune-depression) - Cost-effectiveness study:  R $61,710 would be saved if study population was vaccinated, and complications/death avoided |
| dos Santos *et al.* 2008 | Brazil | 1 city | Cross sectional Retrospective | Questionnaire and serological survey | 2002 | Neonatal units | Healthcare workers, aged 20–64 years | 215 | SP | - SP 99.1%; 0.9% had equivocal immunity - Reported history of varicella, 70% (PPV 100%) - Of 65 people without previous history of varicella, 63 (96.9%) were immune | |
| Giglio *et al.* 2018 | Argentina | Multicenter | Retrospective | Chart review | 2009–2014 | Inpatients, outpatients | Children, mean aged 1–12 years | 150 | Complications and costs pre-vaccination | - One or more complications were experienced by 28.0% of outpatients and 98.7% of inpatients - The most common complications were skin and soft tissue infections, pneumonia, sepsis, cerebellitis, and febrile seizure - Healthcare resource utilization estimates included use of OTC medications (58.7% outpatients, 94.7% inpatients), prescription medications (26.7% outpatients, 77.3% inpatients), tests/procedures (13.3% outpatients, 70.7% inpatients), and consultation with allied health professionals (1.3% outpatients, 32.0% inpatients) - Average duration of hospital stay was 4.9 (95% CI: 4.25.7) days, and average duration of ICU stay was 4.8 (95% CI, 1.6 14.1) days - Total combined direct and indirect cost per varicella case was US $2947.7 (inpatients) and US $322.7 (outpatients) - The overall annual cost of varicella in Argentina for children aged ≤ 14 years in 2015 was estimated at US $40,054,378.00 | |
| Kupek & Tritany, 2009 | Brazil | 1 state (Santa Catarina) | Retrospective | Official epidemiological surveillance data | 1997–2007 | Population | Children aged < 1 to 14 years | 135,311 cases of infection in state | Incidence, morbidity | | - 70% of varicella cases were in children aged < 10 years - Effectiveness of vaccine ranged from 27% to 38% among 4 pediatric age groups - Trend of rising incidence among children aged 1–4 years and (to lesser extent) < 1 year in the wider state (no vaccination), and falling incidence in these groups in state capital (vaccination since 2002 for children up to 2 years) - This pattern not seen in children 5–14 years - Significant 75.5% reduction in incidence post-vaccination for 1–4 year age group - 1 death from complications, in an adult |
| Lafer *et al.* 2005 | Brazil | 5 villages in indigenous national park | Prospective | Questionnaire and serological survey | 2001 | Community (homes) | Indigenous inhabitants, aged < 1 year to 21+ years | 589 (by questionnaire) 224 (by blood samples) | SP | | - Overall SP 80.8% (95% CI: 76%, 86%) - Increase in seropositivity with age (from 25% in infants to 100% at 11–15 years) - Reported varicella history: PPV 98%, NPV 41% |
| Léon-Castañeda *et al.* 2013 | Colombia | 1 department (14 municipalities) | Retrospective | Diagnostic survey | 2008–2012 | General population | Both sexes, all ages | 6115 | Incidence, morbidity | | - 6115 varicella cases in study period (mean 1223/year) - Significant increase in cases from 2008 (78.32/100,000 pop) to 2012 (165.83 cases/100,000pop) - Highest incidence was in a rural municipality in 2009, with 476.5 cases/100,000 pop - 51.64% of cases were males; 31.61% in children < 5 years; 1.1% required hospitalization - There were no deaths |
| Longfield *et al.* 1990 | Puerto Rico | Puerto Ricans in San Antonio, TX | Prospective | Epidemiological and serological survey | 1986–1987 | Educational center for students | Male and female army recruits, average age > 21 years | 810 | SP | | - Overall SP 58% - Attack rates in two outbreaks were 30% and 19%, respectively - Positive ELISA results had 95% positive predictive value of clinical immunity |
| Marcitelli & Ferro Bricks, 2006 | Brazil | 1 city | Cross-sectional | Interview with parents; vaccination record review | 2002–2003 | Daycare centers  (*N* = 44) | Children, aged 6 months to 7 years | 664 | Prevalence, complications | | - History of varicella: overall, 61%; after starting daycare, 53% (of whom 8% were aged  < 1 year) - Main symptoms: exanthema (100.0%), fever (85.4%), anorexia (39.7%), headache (15.2%)Complications in 38 children (5.7%; 95% CI: 3%, 8%); 8 (1.2%) were hospitalized, and  5 (0.7%) had sequelae - 77.9% made medical visits (28.1% more than once) |
| Masuet-Aumatell *et al.* 2013 | Bolivia | 1 region | Cross-sectional | Questionnaire and serological survey | 2010 | Schools | Children  5–16 years | 436 | SP | | - Overall SP 78.2% (95% CI: 74.3- 82.1) - SP numerically higher in: females (82.1%), pre-adolescents (10.1–13 years: 81.4%), adolescents (> 13 years: 80.2%), children with less well-educated parents (81.3%–81.6%), warmer municipalities (81.4%–82.2%), rural areas (80.0%), children with siblings (80.1%) |
| Miranda-Choque *et al.* 2013 | Peru | 1 city | Descriptive-exploratory study | Case records | 2001–2011 | 1 hospital | Both sexes, children | 1073 | Morbidity | | - Hospitalized children with complicated varicella infection - Median age was 2.5 years (IQR: 1.1–4.8 years); 578 (54%) were male - Most frequent complications were secondary skin and soft issue infections with 768 cases (72%) - 13 deaths (1.4%) were recorded |
| Paternina-Caicedo *et al.* 2013 | Colombia | Nationwide | Economic | Serological survey data | 30-years period | General population | Both sexes, all ages | 700,197 varicella cases/year (60 deaths/year) | Cost-effectiveness | | - Estimated burden of disease costs (2008-dated) for all cases during 30-year period:   - Without vaccination: US $88,734,735   - 1-dose: US $174,899,415   - 2-dose:US $307,736,444 - Cost per life-year gained: one-dose vaccination: US $2519; two-dose scheme: US $5728 |
| Quian *et al.* 2004 | Uruguay | 1 city | Retrospective | Serological survey data | 2000 | General population | Both sexes, 1-40 years | 972 | SP | | - Global prevalence of anti-VZV antibodies was 82.1% (798/972) - At age 4 years, 61.9% had already had exposure to the VZV - In multiple logistic regression analysis, age, low social and economic levels, and family history of varicella were independent predictors of SP for varicella |
| Quian *et al.* 2008 | Uruguay | 1 city | Retrospective and prospective | Health surveillance systems | 1997–2005 | General population | Both sexes,  < 1–14 years | 294,831 | Hospitalization rates | | - By 2005 relative to 1997–1999, proportion of hospitalizations due to varicella was reduced by 81% overall (by 63%, 94%, 73%, and 62% in < 1-, 1–4-, 5–9-, and 10–14-years age groups, respectively) - Incidence of ambulatory visits for varicella was reduced by 87% overall (by 80%, 97%, 81% and 65% in < 1-, 1–4-, 5–9-, and 10–14-years age groups, respectively) |
| Ruvinsky *et al.* 2015 | Argentina | 1 city | Descriptive-exploratory study | Database survey | 2008-2013 | Pediatric hospital | Children exposed to varicella in hospital | 107 | Morbidity | | - 29% recalled having had varicella, 27% reported never having the disease, and 41% did not recall a history of varicella - Only 3% had been vaccinated - Based on their immune status, age, and history of varicella, acyclovir was indicated as prophylaxis in 61%, immunization in 10%, and gamma globulin in 1 patient |
| Semenovitch & Lupi, 2003 | Brazil | 1 state | Retrospective | Questionnaire and serological survey | Not stated | 2 university hospitals | Pediatric inpatients, aged 5 months to 12 years | 160 | SP | | - Overall SP, 58.1% - SP increased with: age, to 80%–100% at 10+ years; history of measles; varicella and presence of skin lesions - SP only 83% among those with reported history of varicella - 41% with no varicella history were seropositive |
| Valentim *et al.* 2008 | Brazil | Nationwide | Economic | Serological survey data, hospitalization rates | 30-year period | General population | Both sexes, all ages | 2,915,294 varicella cases/year | Cost-effectiveness | | - Assuming single-dose schedule, vaccine efﬁcacy of 85%, and coverage of 80%, the program could prevent 74,422,058 cases and 2905 deaths - Estimated cost: R $3,178,396,110, with saving of R $660,076,410 to society and R $365,602,305 to healthcare system |
| Vergara-Castañeda *et al.* 2012 | Mexico | Nationwide, divided into 3 climate regions | Retrospective | National surveillance database | 1995–2010 | Reported varicella cases | Both sexes, all ages | National population | Varicella incidence | | - Incidence range from 233 to 381 cases/100,000 during 1995–2010 - Higher incidences of varicella in warmer vs. temperate regions - Young children were the most affected age group - No correlation between varicella incidence and overcrowding or population density |
| Vico & Laurenti, 2004  In Portuguese | Brazil | 1 city | Prospective descriptive | Local databases | 1995–1999 | Municipal daycare center | Children aged < 7 years | Not stated | Mortality | | - Varicella-related deaths: *n* = 12 in daycare, *n* = 48 in municipality of Sao Paulo; death rates 2.40/10^5^ and 1.05/10^5^, respectively |
| Villasís-Keever *et al.* 2001 | Mexico | 1 city | Prospective | Questionnaire and serological survey | 1998 | Pediatric hospital | Medical residents | 89 | SP | | - Vaccination against varicella: 3.3% - History of varicella: 83.1% - Contact with varicella patients: 91.0% - SP: 98.8% - History of prior varicella: PPV, 100%; NPV 7% |
| Yu *et al.* 2000 | Brazil | 1 city | Prospective | Serological survey | 1992–1994 | Public schools | Children, aged 1–15 years | 5343 | SP | | - SP 40% in 1 year olds (33.0% for 1992, 48.5% for 1993, and 35.0% for 1994) - Across the age groups, SP highest in 11 year olds and lowest in 1 year olds in 1992–1993 |
| Yu *et al.* 2001 | Brazil | 2 cities in 1 state | Prospective | Serological survey | 1992 | Public schools and home | Children, aged 1–15 years | 11768 | SP | | - Average age of first infection was 2.87 ± 0.14 years and  4.07 ± 0.47 years for São Paulo and Caieiras, respectively - Non-linear trend of increasing SP with age: in São Paulo and Caieiras city, SP in 1 year olds 33% and 18% respectively, rising to 80.2% and 94.8% in children ≥ 10 years - SP and force of infection were higher in São Paulo schoolchildren up to age 3 years than children in Caieiras, where social contact starts later |

*ELISA* Enzyme-linked immunosorbent assay; *IQR* interquartile range; *NPV* Negative predictive value; *PPV* Positive predictive value; *VZV* Varicella-zoster virus; *SP* seroprevalence
